# Anaerobic digestion of the microalga *Spirulina* at extreme alkaline conditions: biogas production, metagenome, and metatranscriptome

**DOI:** 10.3389/fmicb.2015.00597

**Published:** 2015-06-22

**Authors:** Vímac Nolla-Ardèvol, Marc Strous, Halina E. Tegetmeyer

**Affiliations:** ^1^Institute for Genome Research and Systems Biology, Center for Biotechnology, University of BielefeldBielefeld, Germany; ^2^Department of Geoscience, University of CalgaryCalgary, AB, Canada; ^3^Microbial Fitness Group, Max Planck Institute for Marine MicrobiologyBremen, Germany; ^4^HGF-MPG Group for Deep Sea Ecology and Technology, Alfred Wegener Institute, Helmholtz Centre for Polar and Marine ResearchBremerhaven, Germany

**Keywords:** haloalkaline, biogas, methane rich, microalgae, alkaline lake, *Spirulina*, *Methanocalculus*

## Abstract

A haloalkaline anaerobic microbial community obtained from soda lake sediments was used to inoculate anaerobic reactors for the production of methane rich biogas. The microalga *Spirulina* was successfully digested by the haloalkaline microbial consortium at alkaline conditions (pH 10, 2.0 M Na^+^). Continuous biogas production was observed and the obtained biogas was rich in methane, up to 96%. Alkaline medium acted as a CO_2_ scrubber which resulted in low amounts of CO_2_ and no traces of H_2_S in the produced biogas. A hydraulic retention time (HRT) of 15 days and 0.25 g *Spirulina* L^−1^ day^−1^ organic loading rate (OLR) were identified as the optimal operational parameters. Metagenomic and metatranscriptomic analysis showed that the hydrolysis of the supplied substrate was mainly carried out by Bacteroidetes of the “ML635J-40 aquatic group” while the hydrogenotrophic pathway was the main producer of methane in a methanogenic community dominated by *Methanocalculus*.

## Introduction

Extremophilic microorganisms are bacteria and archaea which inhabit, thrive in and colonize environments characterized by extremely harsh conditions (Berlemont and Gerday, 2011; Gupta et al., 2014). Haloalkaline microorganisms are a specific group of extremophiles which have the ability to thrive at high concentrations of salt, up to 7.0 M and high pH, up to pH 11, and high carbonate concentration (Grant et al., 1990; Baumgarte, 2003; Sorokin et al., 2014). Their specific abilities to withstand high alkalinity and high salinity have pawned multiple biotechnological applications, such as enzymes for the detergent industry and the bioremediation and biotransformation of waste from haloalkaline processes (Horikoshi, 1999; Van Lier et al., 2001; Zhao et al., 2014).

Haloalkaline microorganisms have also been proposed to be applied in the production of biofuels such as hydrogen and ethanol (Zhao et al., 2014). Van Leerdam et al. (2008) showed that it was possible to use a haloalkaline consortium to produce biogas under controlled conditions. The ability of haloalkaline bacteria and archaea to live in alkaline environments could be exploited to produce biogas rich in methane. At alkaline conditions, the carbonate system, CO_2_/HCO^−^_3_/CO^2−^_3_/OH^−^, shifts toward the formation of bicarbonate, CO^2−^_3_, therefore, the CO_2_ released during the decomposition of organic matter (OM) would remain trapped in solution as (bi)carbonate resulting in biogas composed mainly of methane. This methane rich biogas could directly be used as biomethane for vehicles or for the national gas grid (Persson et al., 2006; Weiland, 2010).

In order to produce biogas at alkaline conditions, it is necessary to use a haloalkaline microbial consortium which, for example, can be obtained from soda lakes which are natural ecosystems with pH values of up to 12 and high salt concentrations (Grant, 2006). Some studies have already demonstrated the presence of methanogenic archaea as well as the production of methane in soda lakes and in soda lake sediments (Sorokin et al., 2004, 2015; Nolla-Ardèvol et al., 2012). *Spirulina* is a microalga known to grow in such soda lakes (Jones and Grant, 1999) and has already been used as substrate for biogas production at mesophilic pH conditions (Samson and LeDuy, 1986; Varel et al., 1988; Mussgnug et al., 2010).

Metagenomics has become a common technique to study taxonomy and gene composition in uncultured microbial communities (Simon and Daniel, 2011). The binning of assembled contigs (based on tetranucleotide frequencies) into provisional whole genome sequences can give information about the most abundant and relevant community members (Strous et al., 2012). Moreover, provisional whole genome sequences enable the inference of an ecological function for each major community member (e.g., biomass hydrolysis, fermentation, methanogenesis etc.). Metatranscriptomics, the sequencing and analysis of mRNAs, can give information about the actual active functions of a given microbial community (Gilbert et al., 2008; Urich et al., 2008). The combination of the binning approach, where provisional whole genome sequences of the abundant community members are generated, with the mapping of transcriptome reads to these provisional genomes, can give firsthand information about the ecological function of each of the most abundant organisms present in a microbial community (Chistoserdova, 2014).

The anaerobic digestion of OM is a complex process that involves the participation of both bacteria and archaea (Schlüter et al., 2008; Wirth et al., 2012). Under alkaline conditions this likely also applies but to date, the different functional groups have only been addressed individually (Sorokin and Kuenen, 2005b; Kivistö and Karp, 2011; Antony et al., 2012; Sorokin et al., 2015).

In this work we present, to the best of our knowledge, the first study of biogas production from organic biomass at alkaline conditions (pH ~10; 2.0 M Na^+^) in a semi-continuous stirred tank reactor inoculated with a strict haloalkaline microbial consortium. A good understanding of the taxonomic composition and the functional interactions between the involved microbial populations can contribute to the optimization of the anaerobic digestion of the desired substrate. Therefore, the metagenome and metatranscriptome of the haloalkaline anaerobic community responsible for the degradation of OM and the production of methane is also presented.

## Materials and methods

### Bioreactor set-up

A 2.0 L semi-continuous stirred tank reactor (S-CSTR) with a working volume of 1.5 L operating at 35°C and at high pH (~10) and high salt concentration (2.0 M Na^+^) was set up and operated at anaerobic conditions. The same reactor was used in three different experiments: (i) determination of the optimal Hydraulic Retention Time (HRT) (Alk-HRT); (ii) determination of the optimal Organic Loading Rate (OLR) (Alk-OLR) and (iii) operation at optimal identified parameters (Alk-Opt). The substrate, freeze dried *Spirulina* (Sonnenmacht GmbH, Germany) and the alkaline medium, in g L^−1^: Na_2_CO_3_, 95.0; NaHCO_3_, 15.0; NaCl, 16.0 and K_2_HPO_4_, 1.0; were the same for all three experiments. Two different micronutrients solutions were used throughout the different experiments (Table 1). Solution-1 was used in reactors Alk-HRT and Alk-OLR while Solution-2 was used in Alk-Opt. The medium was prepared in lots of 1.0 L, its pH was adjusted to 10.0 at 35°C, and was stored at 37°C until use. Feed was prepared fresh every day by dissolving the appropriate amount of *Spirulina* in alkaline medium in order to obtain the desired organic loading rate. The daily purge and feed were performed manually with a syringe and through a settler. To avoid excessive loss of microorganisms, the biomass was settled before purging by stopping the stirring for at least 2 h. Periodically the purged sludge was sampled for analysis; in that case the stirring was not stopped. pH and redox potential in the reactors were monitored with a Mettler Toledo pH probe (HA405-DPA-SC-S8/225) and a Mettler Toledo Redox probe (Pt4805- DPA-SC-S8/225) respectively (Mettler Toledo GmbH, Germany). Mesophilic temperature conditions were maintained with a Pt-1000 temperature sensor and a heater.

**Table 1:** **Micronutrient solution composition**.

	Solution-1^*^	Solution-2^**^
Reactors	Alk-HRT/Alk-OLR	Alk-Opt
Compound	mg L^−1^	mg L^−1^
FeSO_4_ · 7H_2_O	–	2000
FeCl_2_ · 4 H_2_O	2000	–
MnCl_2_ · 4H_2_O	500	500
H_3_BO_3_	50	300
ZnCl_2_	50	–
CoCl_2_ · 6H_2_O	–	200
Na_2_SeO_3_ · 5H_2_O	164	164
NiCl_2_ · 6H_2_O	–	92
ZnSO_4_ · 7H_2_O	–	100
AlCl_3_ · 8H_2_O	–	90
(NH_4_)_6_Mo_7_O_24_ · 4H_2_O	50	50
CuCl_2_ · 6H_2_O	38	38
Yeast extract	200	200
Vitamins RPMI-1640^***^		1.0 ml

*Solution was prepared in 1 L batch and added to the macronutrient solution at a concentration of 10 mL per liter*.

* *Modified from Vidal et al. (1997)*.

** *Dr. Dimitry Y Sorokin personal communication*.

*** *Sigma Aldrich*.

### Analytical methods

In addition to continuous measurements of pH and redox potential, alkalinity and total and volatile solids (TS and VS) in the digesters were periodically analyzed. Biogas production was determined by measuring the pressure build up with a pressure-meter (WAL-BMP-Test system 3150, WAL, Germany) and normalizing to standard conditions (0°C; 1.0 atm). Biogas composition (CH_4_, CO_2_, and H_2_S) was analyzed once a week by means of a Shimadzu GC-2010 plus Gas Chromatograph (Shimadzu Corp, Japan) equipped with an Agilent GS-Gaspro capillary column (part # 113-4362) (Agilent Technologies, USA). Samples for biogas composition were obtained using a gas-tight syringe and were kept in 3.0 ml gas-tight vials (Labco Limited, UK) until analysis. Analyses to characterize the digester effluent were carried out periodically directly with the raw sample and with the soluble fraction by centrifuging the samples at 4600 rpm for 5 min and filtering the supernatant through a Rotilabo CME 0.45 μm nylon filter (Carl Roth GmbH, Germany). Once a week, TS and VS were analyzed following the 2540B and 2540E methods of the American Public Health Association (APHA, 2005) standard methods. Alkalinity, OM, measured as total chemical oxygen demand (COD_T_), and ammonium nitrogen (NH^+^_4_-N) were analyzed using colorimetric methods (Hach Lange GmbH, Germany). Soluble COD (COD*_S_*) and total nitrogen (TN) were analyzed once every 2 weeks also with Hach Lange colorimetric methods. Free ammonia nitrogen (NH_3_-N) concentration was calculated as in Astals et al. (2012). Samples for measuring specific volatile fatty acids (acetate, propionate, iso-butyrate, n-butyrate, iso-valerate, and n-valerate) were prepared according to the APHA (2005) 5560D procedure and analyzed using a Shimadzu GC-2010 plus Gas Chromatograph coupled to an FID detector and equipped with a Macherey-Nagel Optima FFA plus capillary column (Macherey-Nagel GmbH & Co. Germany). Theoretical biomethane potential (BMP_Th_) of *Spirulina*, 627 ml CH_4_g VS^−1^, was calculated with Equation (1) based on the chemical composition C_4_H_7_O_1_N_0.8_S_0.02_ (Ortega-Calvo et al., 1993). The percentage of substrate conversion to methane, BD_CH4_(%), was calculated with the Equation (2).

(1) $$BMP_{\text{Th}}=\frac{\left[\left(\frac{a}{2}\right)+\left(\frac{b}{8}\right)-\left(\frac{c}{4}\right)-\left(\frac{3d}{8}\right)-\left(\frac{e}{4}\right)\right]*22,400}{\left(12a+b+16c+14d+32e\right)}$$

                                                       (Raposo et al., 2011)

(2) $$BD_{\text{CH4}}\left(\%\right)=\frac{Bo-Exp}{Bo-Th}*100$$

                                                       (Raposo et al., 2011)

Where, a, b, c, d, and e come from the empirical formulae (C_a_H_b_O_c_N_d_S_e_) and Bo—Exp is the experimental methane production (CH_4_g VS^−1^) and Bo—Th is the BMP_Th_ of *Spirulina*.

### Determination of the optimal hydraulic retention time (HRT)

Reactor Alk-HRT was inoculated with 1,200 ml of alkaline sludge obtained from a start-up alkaline reactor inoculated with a mixture of soda-lake sediments obtained from the Kulunda steppe (Russia) in 2010 and also fed with freeze dried *Spirulina*. Additionally 300 ml of fresh alkaline medium were added resulting in a total volume of 1,500 ml. The reactor was operated with an OLR of 1.0 g *Spirulina* L^−1^ day^−1^ (dry weight) and at five different HRT, 5, 10, 15, 20, and 30 days. An initial 25 days adaptation period was performed during which the purge and feeding of the reactor was done every 2 days at 1.0 g *Spirulina* L^−1^ day^−1^ (dry weight) and with a 20 day HRT. Subsequently the feeding was shifted to daily feeding while the HRT was maintained at 20 days and the experiment started. Table 2 shows the duration of the test periods and the amount of medium exchanged daily for each of the tested HRTs. After 215 days of continuous biogas production the experiment was concluded and the reactor stopped.

**Table 2:** **Alkaline anaerobic reactors**.

	**Anaerobic bioreactors**								
	**Alk-HRT**					**Alk-OLR**			**Alk-Opt**
Periods	**I**	**II**	**III**	**IV**	**V**	**I**	**II**	**III**	–
Duration (Days)	20	25	40	38	92	52	46	41	67
From–To (Days)	1–20	21–44	45–84	85–123	124–215	1–53	54–99	100–141	1–67
HRT (Days)	20	5	10	30	15	15	15	15	15
Purge/Feed (ml day^−1^)	75	300	150	50	100	100	100	100	100
OLR (g *Spirulina* (L_R_ day)^−1^)^*^	1.0	1.0	1.0	1.0	1.0	0.25	0.50	1.0	0.25

*Operational parameters and duration of each different period for the three anaerobic bioreactors used, Alk-HRT, Alk-OLR, and Alk-Opt*.

* *Dry weight*.

### Determination of the optimal organic loading rate (OLR)

One thousand two hundred milliliter of sludge from the Alk-HRT reactor plus 300 ml of alkaline medium were used to inoculate the same S-CSTR, now Alk-OLR. The Alk-OLR reactor was operated at 15 days HRT and at different OLR, 0.25, 0.5, and 1.0 g *Spirulina* L^−1^ day^−1^ (dry weight) (Table 2). Before the experiment was started, the reactor was fed every 2 days and operated at a loading rate of 0.25 g *Spirulina* L^−1^ day^−1^ for a period of 15 days. The Alk-OLR experiment lasted for 141 days after which the reactor was stopped.

### Operation at optimal HRT and OLR conditions

Alk-Opt reactor was operated at the optimal HRT, 15 days, and optimal OLR, 0.25 g *Spirulina* L^−1^ day^−1^ (dry weight). The inoculum for Alk-Opt consisted of 1.5 L of alkaline sludge obtained from a second alkaline reactor (Alk-Sed-2) which was inoculated with a second batch of fresh sediments obtained from the same soda lakes in 2012 and used within 6 months of the sampling date and had been operating for over 190 days with constant biogas production. The start-up of the Alk-Opt reactor consisted of a 15 days period during which *Spirulina* (0.25 g L^−1^ day^−1^) feeding was performed every 2 days with a 15 days HRT. After the start-up period the experiment started and the feeding was set to a daily basis. The reactor was operated for 67 days (Table 2).

### Metagenome and metatranscriptome analysis

#### DNA and RNA extraction

15.0 mL of sludge obtained from reactor Alk-Sed-2 were used for DNA extraction. DNA was extracted according to Zhou et al. (1996) with minor modifications in order to optimize the DNA extraction. The sample was first washed three times with a 1.0 M NaCl solution to reduce the alkalinity. Additionally, during the Lysozyme incubation period, 200 μL of 200 mM AlNH_4_(SO_4_)_2_ solution was added to precipitate humic acids (Braid et al., 2003; Foti et al., 2008). Extracted DNA was purified with an ion exchange column (Macherey-Nagel, Germany) and re-suspended in TE buffer.

RNA was extracted from the same anaerobic reactor 2 days after the DNA extraction. Between 12 and 16 ml of fresh sludge was obtained from the reactor, centrifuged for 10 min at 4°C and 14,000 rpm. Supernatant was removed and for each milliliter of remaining pellet, 3.0 ml of “Life Guard Soil Preservation Solution” (MoBio # 12868-100) (LGPS) were added. The mixture was vortexed, stored 2 days at 4°C to allow preservation of cells and subsequently stored at −20°C until used for RNA extraction. RNA was extracted following Smith et al. (2007) protocol with minor modifications. To reduce alkalinity and salt concentration of the stored samples, a preliminary washing step was performed (5 min centrifugation at 4°C and 14,000 rpm to pellet and remove supernatant, followed by addition of 2.0 ml LGPS and a second centrifugation step). The supernatant was removed and 1.0 ml of TRI reagent was added to the pellet. From this point, the Smith et al. (2007) protocol was followed. 18 μg of total extracted RNA were treated with DNase and Riboblock RNase inhibitor (Fermentas, Thermo Fisher Scientific GmbH, Germany) and purified with Qiagen RNeasy MiniElute Cleanup kit (Qiagen GmbH, Germany). Ribo-zero rRNA removal kit (Bacteria) was used to remove ribosomal RNA from the purified RNA sample. Purified mRNA was stored at −80°C until sequencing library preparation.

#### DNA and cDNA library preparation

2.5 μg of purified extracted DNA were used to prepare a 400 bp insert size sequencing library for the Ion Torrent Personal Genome Machine (PGM) platform (Life Technologies, USA). The instructions according to the Ion Xpress™—Plus gDNA Fragment Library Preparation manual were followed, except for the initial DNA fragmentation, which was done using a GS FLX Standard Nebulizer Kit (Roche Applied Science, Germany), nebulization for 3 min at 32 psi.

For cDNA library preparation, the “Ion Total RNA-seq kit v2 for whole transcriptome libraries” was used. 20 ng mRNA of the sample were used to prepare the cDNA library. Sequencing template preparation was performed using the OneTouch Instrument. Enriched ISP particles were sequenced with the Ion PGM™ 400 Sequencing Kit (Life Technologies, USA) on a 318™ Chip with 1,000 flows for both the DNA and cDNA sequencing, following the manufacturer's instructions.

Automated quality control of the sequenced reads was performed with the Torrent Suite™ Software v3.2 using default settings. Additional quality filtering was done using the Trimmomatic tool v3 (http://www.usadellab.org/cms/index.php?page=trimmomatic) (Lohse et al., 2012) with settings for removal of trailing bases of a *q*-value lower than 20, and removal of remaining reads shorter than 100 bp and longer than 450 bp for DNA and shorter than 20 bp and longer than 350 bp for cDNA.

### Metagenome analysis

#### Removal of *Spirulina* reads

Quality trimmed reads were uploaded to the MGX platform, a metagenomics platform currently being developed at the CeBiTec (Bielefeld University), for a rapid prescreening of the reads. A preliminary taxonomic analysis revealed over 30% of reads assigned to *Spirulina* which were removed as follows: First, 3,125 sequences, from all genomes and curated sequences (RefSeq) from the NCBI database (November 2013) classified as *Spirulina*/*Arthrospira*, were downloaded. Next, quality trimmed reads were blasted (Altschul et al., 1990) against these 3,125 sequences in order to identify reads originating from *Spirulina*. Blastn was performed with an *e*-value of 1e-10, a maximum of one target per sequence and with a minimum of 98% of identity. Any read that had a blast hit to any of the downloaded *Spirulina* sequences was removed from the dataset.

#### Analysis of sequencing reads

##### Analysis of assembled reads

Quality trimmed, *Spirulina* filtered reads were assembled into contigs using the Genome Sequencer De Novo Assembler Software v2.8 (Roche Applied Science, Germany). Two assemblies (A and B) were performed: assembly A was done with default settings for genomic DNA, and assembly B was done with more stringent settings, according to Fan et al. (2012), for better assembly of 16S rRNA sequences.

Contigs from assembly A were binned into provisional whole genome sequences of abundant populations in order to taxonomically analyze the microbial population. Contigs were binned, using the Metawatt v1.7 pipeline (http://sourceforge.net/projects/metawatt) (Strous et al., 2012). Binning options were set as follows: read length 200 nt; minimum bin size 100 kb and minimum contig size 500 bp. Generated bins were manually revised and assigned to a taxon by blasting all contigs from the selected bins against the 16S rRNA SILVA database (Quast et al., 2013). Coverage and bin size of each particular bin were used to estimate the abundance of each population. Furthermore, transfer-RNAs of each bin were identified with ARAGORN (Laslett and Canback, 2004) and the genome completeness for each population was estimated by the identification of 139 conserved Pfams as described by Campbell et al. (2013).

##### Phylogeny of assembled 16s rRNA sequences

To identify 16S rRNA sequences among the assembled contigs, all contigs from assemblies A and B were submitted to a blastn search against the RDP database (v11-2) (Cole et al., 2013). Sequence parts with a hit were extracted and aligned parts with a minimum length of 1,000 bp for bacteria and archaea and 500 bp for Bacteroidetes were used to create phylogenetic trees.

The assembled 16S rRNA sequences were submitted both to the RDP classifier (Wang et al., 2007) and the SINA classifier (Pruesse et al., 2012) with the confidence threshold or minimum sequence similarity set to 80%, respectively. The sequences were also submitted to a blastn search against the May 2014 NCBI nucleotide collection (nr/nt), and reference RNA sequences (refseq_rna). For both blastn searches the top blast hit for each query sequence was obtained. All sequences (16S rRNA contig parts and blast search hits), were aligned with muscle (Edgar, 2004). Phylogenetic trees were generated with FastTree (Price et al., 2010) with the GTR+CAT model, bootstrapping (500 reps.) was done using seqboot [v3.67, http://evolution.genetics.washington.edu/phylip.html (Felsenstein, 2005)]. Bootstrap values were implemented into the main tree using the CompareToBootstrap.pl script (Price M. N., http://www.microbesonline.org/fasttree/treecmp.html). Finally, trees were drawn using Dendroscope (Huson et al., 2007).

### Metatranscriptome analysis

#### Functional annotation of selected bins

Selected bins generated as described in (Section Analysis of Assembled Reads) were functionally annotated. First, all contigs from a given bin were concatenated into one super-contig using the linker sequence CTAGCTAGCTAG. Each super-contig was uploaded to GenDB and automatically annotated (Meyer et al., 2003). The same procedure was done with all contigs that were not assigned to any bin, UnbinnedContigs.

#### Mapping of transcripts

Bowtie2 v2.2.4 (Langmead and Salzberg, 2012) was used to align quality trimmed transcript reads to each annotated super-contig. To ensure that a particular transcript was only aligned once, first, all super-contigs, including the UnbinnedContigs were combined into one single file. Subsequently Bowtie2 was used to create indexes from the combined super-contigs and the mapping of transcripts was done using the–very-sensitive option (see http://bowtie-bio.sourceforge.net/bowtie2/manual.shtml#setting-function-options for details).

#### Analysis of transcript mappings

Genbank files containing the CDS sequences obtained with GenDB, and SAM files generated with Bowtie2 were uploaded to ReadXplorer for analysis of the mapped transcripts (Hilker et al., 2014). The “RPKM” option with default settings was used to determine the expression of the identified CDS in each super-contig. The “Feature Coverage Analysis” tool was used to remove those CDS with mapped mRNA transcripts with less than 1× mean coverage and covered less than 50% of the given CDS. In some particular cases, CDS mapped with transcripts were not assigned to any function by GenDB. In this case, the 10 most active CDS according to their RPKM value were blasted against the protein RefSeq database (April 2015) and the top hit was assigned as the CDS function. For comparison between different bins, the RPKM values were normalized to the assembly depth (sequencing coverage) of each bin.

#### Detection of genes and transcripts specific for methanogenesis

All DNA sequences assigned to the three different methanogenic pathways (modules M00567, M00356, and M00357) were downloaded from the KEGG database (Kanehisa and Goto, 2000) in April 2015 and blasted (blastn; cutoff *e*-value 3e-03) against the CDS sequences obtained with GenDB from the methanogen super-contig and against all unbinned contigs. RPKM value of the mapped mRNA transcripts was used to evaluate the activity of the identified enzymes.

Metagenomic and metatranscriptomic reads and assembled contigs are accessible via NCBI under the Bioproject PRJNA281982. The Whole Genome Shotgun project has been deposited at DDBJ/EMBL/GenBank under the accession numbers LCWY00000000 and LCWZ00000000. The versions described in this paper are versions LCWY01000000 and LCWZ01000000. The sequenced reads were submitted to the Sequence Read Archive with sample accession numbers SRS923957 for the metagenomic reads and SRS923955 for the metatranscriptomic reads. The 17 16S rRNA sequences used for the generation of the phylogenetic trees were submitted to GenBank under the sample number SAMN03565345, with accession numbers KR476494 to KR476510.

## Results

In this work, the anaerobic digestion of the microalgae *Spirulina* at alkaline conditions, pH ~ 10, 2.0 M Na^+^, 60–95 g CaCO_3_ L^−1^, was studied in combination with the analysis of the metagenome and metatranscriptome of the anaerobic haloalkaline microbial community.

### Biogas rich in methane

**Figure 1:**
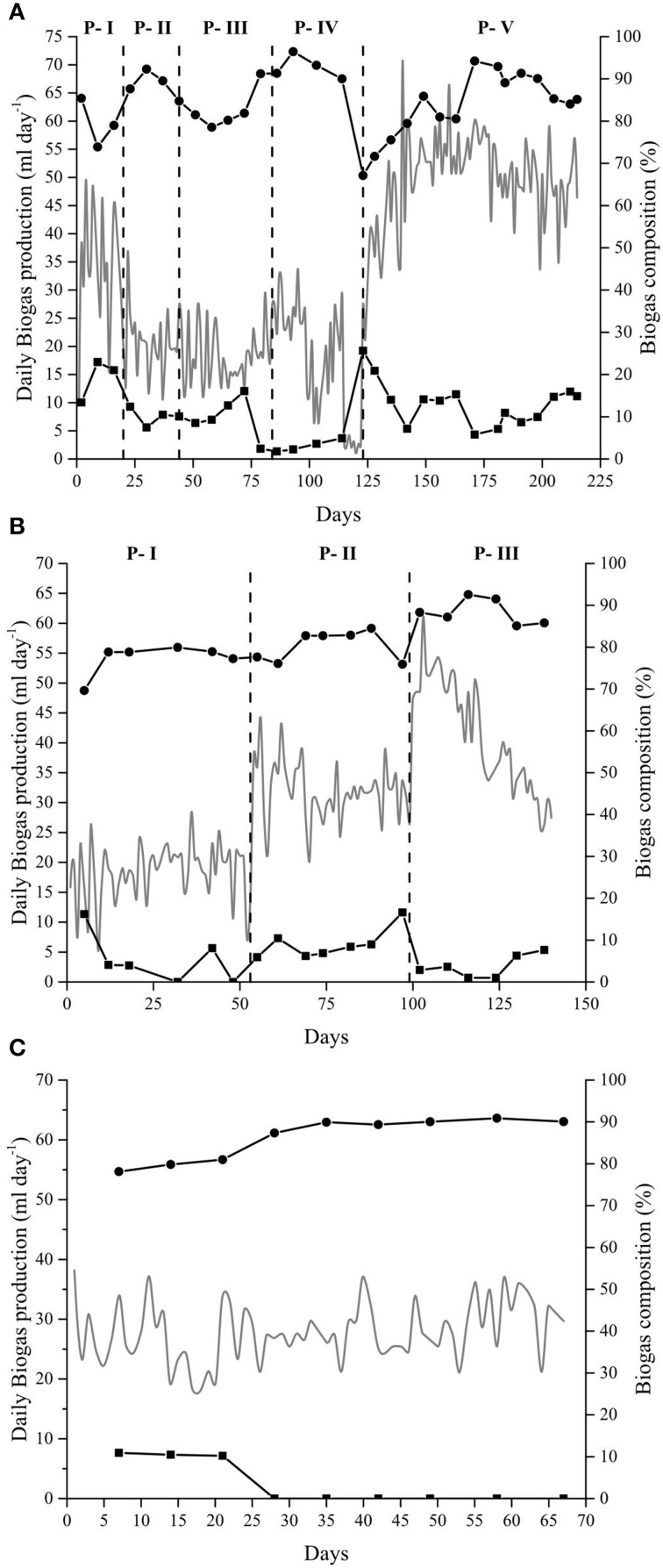
**Biogas production and composition**. Daily biogas production (gray line—left axis) and biogas composition (right axis): CH_4_(•) and CO_2_ (◼), from the anaerobic digestion of *Spirulina* at alkaline conditions in: **(A)** Alk-HRT reactor. Dashed vertical lines indicate a change in the hydraulic retention time: 20 (P-I), 5 (P-II), 10 (P-III), 30 (P-IV), and 15 days (P-V); **(B)** Alk-OLR reactor. Dashed vertical lines indicate a change in the organic loading rate: 0.25 (P-I), 0.5 (P-II) and 1.0 g *Spirulina* L^−1^ day^−1^ (P-III), and **(C)** Alk-Opt reactor. In all three cases, the remaining percentage of gas to reach 100% corresponds to nitrogen gas.

The anaerobic digestion at alkaline conditions produced, as expected, biogas rich in methane throughout the different experiments. In the Alk-HRT reactor, the composition of the biogas was not constant and varied with the changes in the HRT. The mean percentage of methane throughout the experiment was 83% while the carbon dioxide content was 12% (Figure 1A). The highest methane content was obtained in P-IV, day 93, with 96% CH_4_ and in P-V, day 171, with 94% CH_4_, while the CO_2_ content on these 2 days was 2 and 5% respectively. The drop in methane percentage on day 123 (P-IV) was due to a maintenance opening of the reactor. From this day on, the methane content rapidly increased from 67 to 86% and then further to 94% on day 171. At the same time, the carbon dioxide in the headspace gradually decreased (Figure 1A).

The biogas produced in the Alk-OLR reactor was also rich in methane, with a mean value of 82% throughout the three periods (Figure 1B). The highest methane peak was obtained on day 116 with 92% of CH_4_. Carbon dioxide present in the headspace varied between 16 and 1% with its mean value at 6%. As in the other two reactors, methane content in Alk-Opt reactor was also high, with peaks up to 90% and the CO_2_ was practically absent from the headspace of the bioreactor (Figure 1C). It is worth noting that in all three experiments, Alk-HRT, Alk-OLR and Alk-Opt, H_2_S gas was never detected in the headspace of the reactors (Figure 1).

### Determination of the optimal hydraulic retention time (HRT)

In Alk-HRT reactor, five different HRT were tested, 20, 5, 10, 30, and 15 days, periods P-I to P-V respectively (Table 2). In the course of the experiment the HRT was adjusted to improve the biogas production rate: it was reduced when accumulation of potentially harmful ammonia and volatile acids was observed, and it was increased when the concentration of these compounds was low and biomass washout was more likely to be the cause of reduced biogas production. The initial 20 days HRT was chosen based on our experience with the anaerobic digestion of *Spirulina* at neutral pH. Biogas production in Alk-HRT reactor was continuous and the produced biogas was rich in methane (Figure 1A).

Changes in the HRT had a clear effect on the daily biogas production (Figure 1A). Changing the HRT from 20 to 5 days (P-I to P-II) resulted in a decrease in the daily biogas production while doubling the HRT to 10 days (P-III) did not result in a marked increase in the biogas production (Figure 1A). Increasing the HRT to 30 days (P-IV) initially led to an increase in the biogas production, however, on day 99 (day 14 of period P-IV), a sudden drop from 27 to 11 ml of gas per day was observed. In the subsequent days, the daily biogas production gradually recovered until day 115 when it dropped to 1.9 ml (Figure 1A). After 2 days of almost zero biogas production, accumulated potentially inhibitory substances were removed by pausing the *Spirulina* feeding and by replacing 50 ml of sludge each day with fresh alkaline medium. After 5 days the feeding was resumed at 1.0 g *Spirulina* L^−1^ day^−1^ and the HRT was set to 15 days (P-V). The 5 days exchange of sludge for fresh medium had a positive effect and the daily biogas production was resumed and increased from 27 ml (day 123) to 60 ml of biogas per day (day 162) during period P-V. From this point forward, the biogas production was stable at around 50 ml of gas per day until the end of the experiment (Figure 1A).

From the five different HRT tested, 15 days appeared to be the optimal as with this HRT, the highest biogas production and the highest percentage of substrate conversion to methane were achieved (Table 3).

**Table 3:** **Biogas production and biodegradability of** ***Spirulina***.

**Reactor**	**Period**	**HRT (days)**	**OLR (g L^−1^ day^−1^)**	**Biogas (ml biogas day^−1^)**	SBP^*^ mlbiogas (day g VS)^−1^	**CH_4_ (%)**	**CO_2_ (%)**	BD_CH4_ (%)^**^
Alk-HRT	I	20	1.0	35 ± 9	26 ± 7	79 ± 6	19 ± 5	3
	II	5	1.0	21 ± 5	15 ± 4	89 ± 3	10 ± 2	2
	III	10	1.0	18 ± 6	14 ± 4	81 ± 7	12 ± 7	2
	IV	30	1.0	17 ± 10	13 ± 8	86 ± 13	9 ± 8	2
	V	15	1.0	50 ± 8	37 ± 6	83 ± 9	14 ± 6	5
Alk-OLR	I	15	0.25	18 ± 5	56 ± 15	77 ± 4	5 ± 6	7
	II	15	0.50	32 ± 5	48 ± 7	80 ± 4	9 ± 4	6
	III	15	1.0	40 ± 9	31 ± 7	88 ± 3	3 ± 3	4
Alk-Opt	–	15	0.25	27 ± 4	84 ± 14	86 ± 5	4 ± 3	11

*Daily biogas production, Specific Biogas Production and biodegradability of Spirulina obtained in each period of the different alkaline anaerobic reactors*.

* *SBP, Specific biogas production per VS added*.

** *Percentage of biodegradability calculated as in Raposo et al. (2011) and based on the theoretical methane content of Spirulina: 627 ml CH_4_ g VS^−1^*.

### Determination of the optimal organic loading rate

The optimal OLR was determined with alkaline reactor Alk-OLR which was operated at 15 days HRT. The starting OLR was set to 0.25 g *Spirulina* L^−1^ day^−1^ and was gradually increased until reactor failure. As in Alk-HRT, the pH was constant at pH 10 and the alkalinity high. As expected, an increase in the OLR led to an increase in the biogas production (Figure 1B). Increasing the OLR from 0.5 to 1.0 g *Spirulina* L^−1^ day^−1^, however, eventually had a negative effect on the biogas production. After an initial rise in biogas production to 60 ml per day a gradual decrease to 30 ml per day was observed (Figure 1B). Two strategies for removal of inhibitory substances and recovery of biogas production were applied but were unsuccessful to recover the biogas production (data not shown). At this point the reactor was stopped.

From the three different OLR, setting the OLR to 0.25 g *Spirulina* L^−1^ day^−1^resulted in the highest Specific Biogas Production (SBP), 26 ml biogas g VS^−1^, as well as the highest *Spirulina* conversion to methane 7% (Table 3).

### Biogas production at optimal operational parameters

Alk-Opt was operated with the optimal HRT, 15 days and the optimal OLR, 0.25 g *Spirulina* L^−1^ day^−1^ identified with reactors Alk-HRT and Alk-OLR. As can be seen in Figure 1C, constant biogas production was obtained during the 67 days that the reactor was operative and the methane content in the headspace was extremely high. Ammonia (NH_3_), total and soluble OM (measured as COD_T_ and COD_S_) and acetic and propionic acid remained under controlled levels and did not accumulate throughout the experiment.

### Parameters affecting the biogas production

Several parameters such as free NH_3_, VFAs, and OM affected the biogas production in reactors Alk-HRT and Alk-OLR.

**Figure 2:**
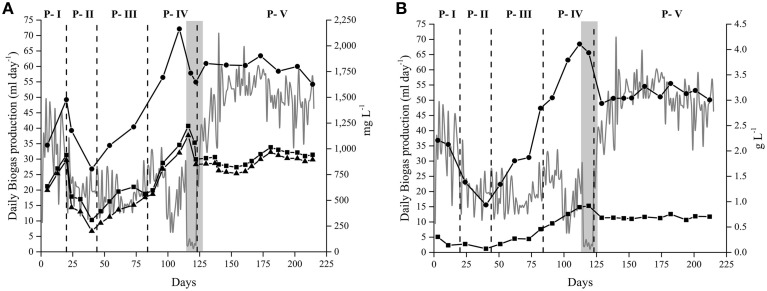
**Parameters affecting the anaerobic digestion of** ***Spirulina*** **at alkaline conditions in the Alk-HRT reactor**. Daily biogas production (gray line—left axis). **(A)** Nitrogen profile (right axis): Total Nitrogen (•), Total Ammonium Nitrogen (TAN) (◼), and Free Ammonia Nitrogen (NH_3_) (▴). **(B)** Volatile fatty acids profile (right axis): Acetic (•) and propionic (◼) acid. Dashed vertical lines indicate a change in the hydraulic retention time: 20, 5, 10, 30, and 15 days. Gray area corresponds to the 5 day non-feeding period.

Changes in the HRT, and therefore in the amount of sludge exchanged daily, had a clear effect on the levels of free NH_3_, and VFAs present in the Alk-HRT medium (Figure 2). A gradual accumulation of these compounds occurred, especially in P-IV (30 days HRT). At the end of this period, the NH_3_ reached 1200 mg L^−1^ (Figure 2A), a concentration much higher than the previously reported inhibitory thresholds, between 150 and 900 mg L^−1^ (Angelidaki and Ahring, 1993; Calli et al., 2005). At the same time, the acetic acid concentration reached its maximum, over 4.0 g L^−1^ (Figure 2B). This accumulation of VFA, but especially of NH_3_, resulted in a sharp drop of the daily biogas production in Alk-HRT due to inhibition of the anaerobic microbial community. To recover the biogas production it was necessary to reduce the levels of inhibitory substances present in the reactor's sludge by stopping the feeding and gradually replacing sludge for fresh medium [see Section Determination of the Optimal Hydraulic Retention Time (HRT)]. This approach reduced the free NH_3_ content to 850 mg L^−1^, as well as the concentration of VFAs, allowing the anaerobic microbial community to recover and for the biogas production to resume (Figure 2). By setting the HRT to 15 days the accumulation of NH_3_, and VFAs was prevented and the inhibitory effect reduced, which resulted in a stable biogas production.

**Figure 3:**
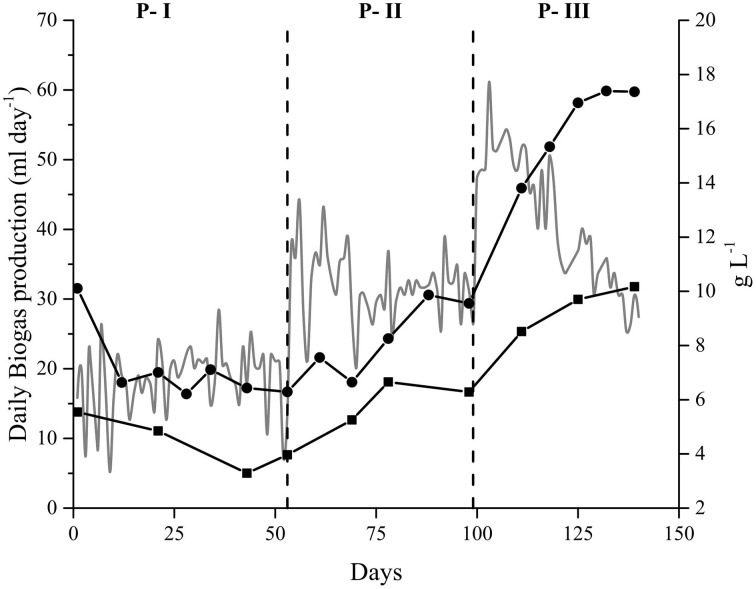
**Parameters affecting the anaerobic digestion of** ***Spirulina*** **at alkaline conditions in the Alk-OLR reactor**. Organic matter profile (right axis): Total (•) and Soluble (◼) Chemical oxygen demand. Dashed vertical lines indicate a change in the organic loading rate: 0.25, 0.5, and 1.0 g *Spirulina* L^−1^ day^−1^. Daily biogas production (gray line—left axis).

The biogas production in the Alk-OLR was influenced by the accumulation of OM (measured as COD_T_ and COD_S_) which drastically affected its performance (Figure 3). When the OLR was increased to 1.0 g *Spirulina* L^−1^ day^−1^, the COD_T_ rapidly increased, from 10 to 17 g O_2_ L^−1^. A similar trend was observed for the soluble organic matter (COD_S_) which increased from 6 to 10 g O_2_ L^−1^. This accumulation of both OM, in the form of COD_T_ and COD_S_, lead to the inhibition of biogas production due to substrate overload (Figure 3).

In contrast to what was observed in Alk-HRT, in Alk-OLR free NH_3_ and VFAs did not reach levels as high as in the Alk-HRT reactor. The NH_3_ concentration in Alk-OLR reached its maximum, 0.73 g L^−1^, in P-III when 1.0 g *Spirulina* L^−1^ day^−1^ was fed as substrate while the concentrations of VFAs remained low throughout the experiment with a slight increase in P-III when 1.0 g *Spirulina* L^−1^ day^−1^ was fed.

**Figure 4:**
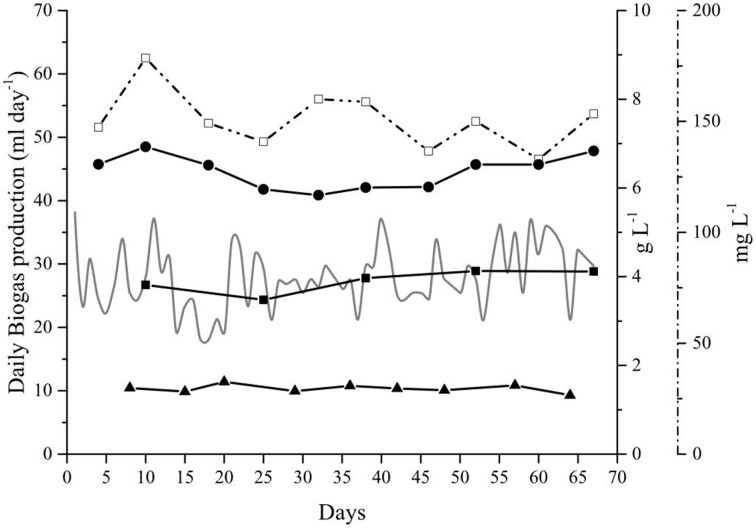
**Main sludge components from the anaerobic digestion of** ***Spirulina*** **at alkaline conditions in the Alk-Opt reactor**. Daily biogas production (gray line—left axis). Free Ammonia (NH_3_) (◻); Total (•) and Soluble (◼) Chemical Oxygen Demand, and acetic acid (▴) profiles (right axis).

When the reactor was operated under optimal conditions, Alk-Opt, no accumulation of NH_3_, VFAs, or OM was observed during the 67 days of operation. The three parameters remained under controlled levels throughout the experiment and did not affect the biogas production (Figure 4).

### Binning and 16S rRNA taxonomy analysis of assembled contigs

DNA was extracted from a running alkaline reactor (Alk-Sed-2) on day 111 of operation (70 mL biogas day^−1^; 92% methane). Quality trimmed reads were assembled in two different assemblies, A and B, which were used for the detection of ribosomal 16S genes to taxonomically characterize the microbial community (see Materials and Methods for details). Assembly A produced longer and fewer contigs due to the more stringent settings of assembly B (Supplementary Table 1). The same 16S rRNA gene sequences were all assembled in each of the two assemblies. Assembly A yielded a higher number of 16S sequences longer than 1,000 bp. However, assembly B produced the longest 16S sequences assigned to Bacteroidetes. The 16S sequences assigned to Methanomicrobiales where identical in length in both assemblies. Assembly A was selected for binning using the Metawatt v1.7 pipeline to investigate the most abundant populations of the microbial consortium in more detail.

Automatic binning yielded nine good bins that contained provisional genomes of abundant populations, based on consistent phylogenetic profiles, presence of a complete set of encoded tRNA molecules and presence of a near complete set of conserved single copy genes. Fragments of 16S rRNA sequences were assigned to each of the bins based on correlation of the phylogenetic profile of the bins with the phylogenetic affiliation of the identified 16S sequences (Table 4). The nine identified bins recruited 60% of the assembled contigs, indicating that together these populations accounted for 60% of the microbial community present in the alkaline reactor on day 111. Bacteria clearly dominated with eight of the nine bins representing over 95% of the total binned populations, while only one of the binned populations belonged to Archaea. Among the Bacteria, Firmicutes and Bacteroidetes were dominant, representing 28 and 27% of the total microbial community respectively, while methanogenic Archaea represented 4.5% of the total microbial community.

**Table 4:** **Selected microbial bins**.

**Bin characteristics**									
**Bin**	**Contigs (#)**	**Size (Mb)**	**N50 contig length (kb)**	**GC (%)**	**Cov (X)**	**tRNA (#)**	Conserved Genes^*^ (#)	**Abun (%)**	16S rRNA taxonomic classification^**^
A	703	2.6	7.3	49.6	21.2	32	128/139	21.2	Bacteroidetes
B	334	1.9	23.1	42.4	9.0	11	103/139	11.1	Clostridiales
C	1900	2.2	1.8	36.9	5.0	24	164/139	7.4	Halobacteroidaceae
D	2851	1.6	0.6	35.2	2.2	4	38/139	2.3	Halanaerobiaceae
E	2693	2.9	1.6	51.2	3.2	9	111/139	6.0	Bacteroidetes
F	2217	2.4	1.9	51.0	2.8	30	97/139	4.5	*Methanocalculus*
G	6796	3.7	0.6	38.6	2.1	43	232/139	5.1	Haloanaerobiales
H	1750	1.2	0.7	46.5	2.9	7	20/139	2.2	Clostridiales
I	1671	0.8	0.5	61.8	2.3	3	55/139	1.3	Rhodobacteraceae

*Characteristics and 16S rRNA taxonomical classification of the nine selected bins obtained from the alkaline metagenome*.

*Cov, Coverage*.

*Abun, Abundance*.

* *Number of Conserved Single Copy Genes detected (out of a set of 139). Numbers higher than 139 indicate the presence of DNA originating from more than a single population in the bin. Numbers lower than 139 indicate the provisional genome sequence associated with the bin may be incomplete*.

** *Taxonomical assignment based on the SILVA and RDP maximum coincidence level. See Supplementary Table 2 for assignment details*.

**Figure 5:**
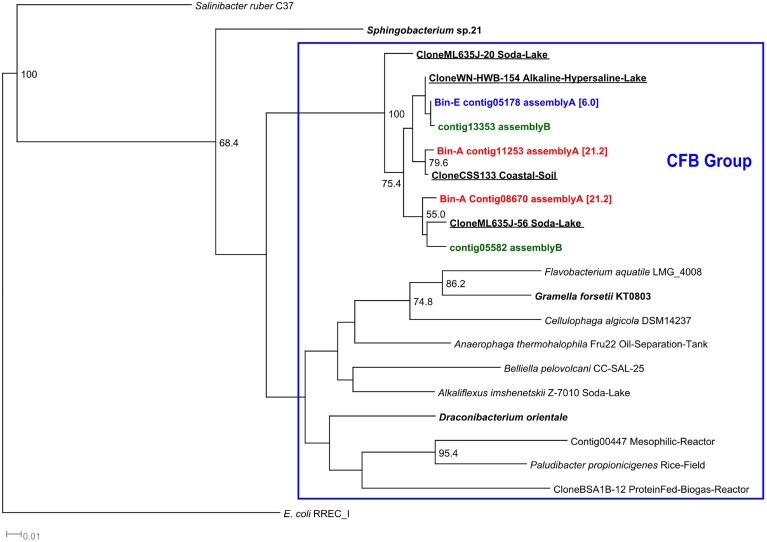
**16S rRNA Cytophaga-Flavobacterium-Bacteroides phylogenetic tree**. 16S rRNA phylogenetic tree of the contigs assigned to members of the Cytophaga-Flavobacteria-Bacteroides group (CFB) by the RDP and SILVA classifiers. Although, the binning was performed with contigs of assembly A, the tree also includes those contigs that were obtained from assembly B and were not assembled in assembly A. Minimum contig length of 500 bp. Colored: sequences obtained from metagenomic reads. Assignment to Metawatt bins and percentage of bin abundance is indicated if applicable. Reference sequences in **bold:** top hits in blast search against NCBI reference RNA sequences database; **bold *+* underlined***:* top hits in blast search against NCBI non-redundant nucleotide collection. Additional reference sequences tree represent genera detected in other alkaline environments or anaerobic digesters. 16S rRNA sequence of *E. coli RREC_I* was chosen as outgroup. Bootstrap values at nodes are obtained from 500 replicates and are only shown for branches with at least 50% support (values > 49.9). The scale bar represents 0.01 nucleotide substitutions per site. Accession numbers of reference sequences are available in Supplementary Table 4.

Members of the Bacteroidetes phylum were the most abundant populations accounting for 27% of the total abundance (Table 4). Bin A (21% abundance) contained three contigs encoding 16S rRNA sequences, which were all assigned to “ML635J-40 aquatic group” by the SILVA classifier and to Bacteroidetes Incertae Sedis, Flavobacteria and Bacteroidia by the RDP classifier, all members of the Cytophaga-Flavobacterium-Bacteroidetes group (CFB) (Supplementary Table 2). Phylogenetically, the three 16S sequences were closely related to several uncultured Bacteroidetes identified in soda lakes (Figure 5). A similar result was obtained in bin E (6% abundance) which was also classified as “ML635J-40 aquatic group” by the SILVA and to Flavobacteria by RDP. Phylogenetically, the 16S sequences were also closely related to uncultured haloalkaline Bacteroidetes.

**Figure 6:**
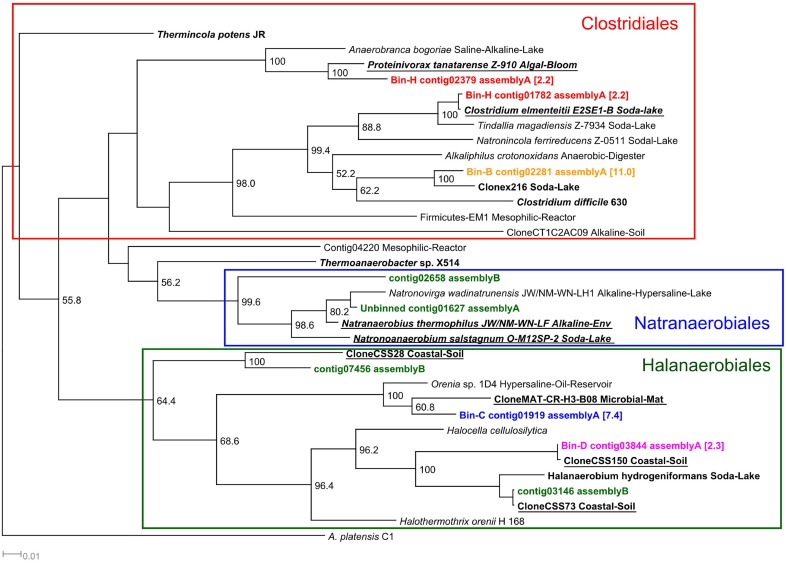
**16S rRNA Firmicutes phylogenetic tree**. 16S rRNA phylogenetic tree of the contigs assigned to members of the Firmicutes phyla by the RDP and SILVA classifiers. Although, the binning was performed with contigs of assembly A, the tree also includes those contigs that were obtained from assembly B and were not assembled in assembly A. Minimum contig length of 1,000 bp. Colored: sequences obtained from metagenomic reads. Assignment to metawatt bins and percentage of bin abundance is indicated if applicable. Reference sequences in **bold**: top hits in blast search against NCBI reference RNA sequences database; **bold + underlined:** top hits in blast search against NCBI non-redundant nucleotide collection. Additional reference sequences tree represent genera detected in other alkaline environments or anaerobic digesters. 16S rRNA sequence of *Arthrospira platensis* was chosen as outgroup. Bootstrap values at nodes are obtained from 500 replicates and are only shown for branches with at least 50% support (values > 49.9). The scale bar represents 0.01 nucleotide substitutions per site. Accession numbers of reference sequences are available in Supplementary Table 4.

The second most abundant group of bacteria were the Halanaerobiales (bins, C, D and G), which represented 15% of the binned microbial community (Table 4). The 16S rRNA sequences detected in the three bins were classified mainly as Halanaerobiales by both classifiers (Supplementary Table 2). The two 16S sequences long enough (>1,000 bp) to be included in the phylogenetic tree (bin C contig01919 and bin D contig03844) were closely related to other uncultured Firmicutes and Halanaerobiales identified in hypersaline or alkaline environments (Figure 6). Clostridiales (bin B and bin H) accounted for 13% of the binned community (Table 4). 16S rRNA sequences present in these two bins were assigned to the Clostridiales order by both classifiers (Supplementary Table 2). Phylogenetically, the detected 16S sequences were closely related to other Clostridiales isolated from several soda lakes and algal blooms (Figure 6). Alphaproteobacteria were also detected in the alkaline anaerobic reactor but their abundance was low, 1.3% (Table 4). Bin I was assigned by both classifiers to the purple non-sulfur bacteria *Rhodobaca* of the family Rhodobacteraceae (Supplementary Table 2). Phylogenetically, the 16S sequence was closely related to *Roseinatronobacter* sp. MOL1.10 identified in Mono lake and an uncultured bacterium, clone TX4CB_152, identified in a highly alkaline and saline soil (Valenzuela-Encinas et al., 2009) (Supplementary Figure 2).

**Figure 7:**
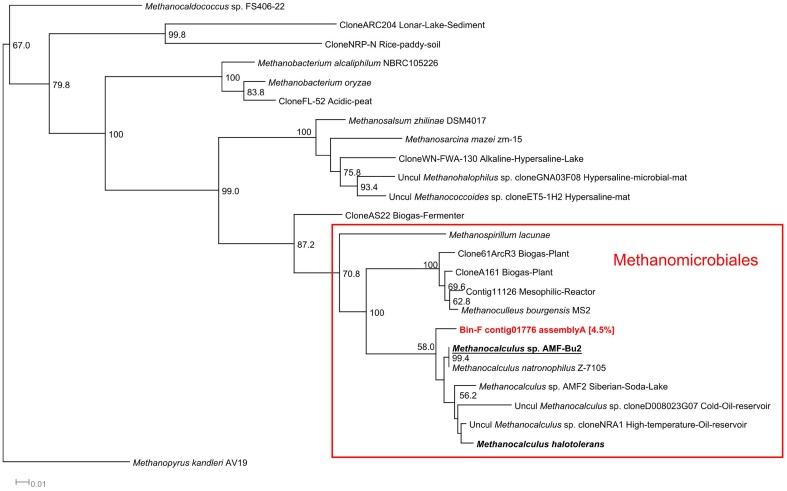
**16S rRNA Methanogens phylogenetic tree**. 16S rRNA phylogenetic tree of the contigs assigned to methanogenic archaea by the RDP and SILVA classifiers. Minimum contig length of 1,000 bp. Colored: sequences obtained from metagenomic reads. Assignment to Metawatt bins and percentage of bin abundance is indicated if applicable. Reference sequences in **bold**: top hits in blast search against NCBI reference RNA sequences database; **bold + underlined:** top hits in blast search against NCBI non-redundant nucleotide collection. Additional reference sequences tree represent genera detected in other alkaline environments or anaerobic digesters. 16S rRNA sequence of *M. kandleri* was chosen as outgroup. Bootstrap values at nodes are obtained from 500 replicates and are only shown for branches with at least 50% support (values > 49.9). The scale bar represents 0.01 nucleotide substitutions per site. Accession numbers of reference sequences are available in Supplementary Table 4.

In the alkaline anaerobic reactor, a single population of methanogens, *Methanocalculus*, a Methanomicrobiales, dominated among the archaeal community (Table 4 and Figure 7). Bin F contained one 16S rRNA sequence which was classified by both classifiers as *Methanocalculus* (Supplementary Table 2). Phylogenetically, this sequence was closely related to *Methanocalculus* sp. AMF-Bu2, identified in sediments from soda lakes of the Kulunda Steppe (Altai, Russia), the same lake system from which the inoculum for the alkaline reactor was obtained, and to *Methanocalculus natronophilus*, isolated from sediments of soda lakes of the Tanatar II system, also in the Kulunda region (Zhilina et al., 2013) (Figure 7).

### Functional analysis of the selected bins

RNA was extracted from the alkaline reactor Alk-Sed-2 on day 113. Metatranscriptome sequencing statistics are presented in Supplementary Table 3. mRNA transcripts were mapped to all nine bins. From these, bins A, B, E, H, and I contained active coding DNA sequences (CDS) which were automatically annotated by GenDB and assigned to different functions. The remaining bins, C, D, F, and G, also contained active CDS however, the automatic annotation by GenDB failed to assign a specific function to the identified CDS (Supplementary Table 3).

Table 5 contains a selection of the most relevant proteins detected in bins A (Bacteroidetes), B (Clostridiales), E (Bacteroidetes), and H (Clostridiales) clustered into three main groups, CDS related to transport functions, CDS involved in general metabolism functions and CDS assigned to functions related to DNA and RNA metabolism. All four bins contained multiple transport enzymes such as ABC transporters, amino acid transporters and TonB-system transporters, all involved in the uptake of substrates, solutes and other metabolites. Several enzymes responsible for the uptake of betaine and choline, both osmoprotectant molecules were also identified among bins A and E (Table 5). It is also worth noting that these same bins also contain multiple Na^+^, Ca^+^, K^+^, and H^+^ cations importers. Among the detected active CDS assigned to general metabolic functions, multiple peptidases and oligopeptidases were identified in bins A, E, and H. Multiple NAD/NADH related proteins were also active among bins A and E. Other enzymes related to general metabolic functions such as glucose metabolism, pyruvate related enzymes and ATPase, synthases and similar, were also detected among the four bins (Table 5). Among the active CDS of bins A, B, and E multiple enzymes related to the DNA/RNA metabolism such as DNA and RNA polymerases were detected (Table 5).

**Table 5:** **Detected active CDS**.

**Enzyme**	**Organism**	**Bin**	**CDS (#)**	**RPKM value**	**Mean % of CDS mapped**
**TRANSPORT**					
ABC transport and related	Bacteroidetes	A	10	135	64
		E	8	864	82
Oligopeptide/dipeptide transport	Bacteroidetes	E	2	119	68
	Clostridiales	B	1	902	67
		H	1	306	66
Amino acid transport and related	Bacteroidetes	A	1	11	62
Other transport enzymes	Bacteroidetes	A	2	151	77
		E	1	70	60
Substrate binging proteins	Clostridiales	B	1	91	60
Glycine/Betaine/Choline transporter	Bacteroidetes	A	3	395	90
		E	4	5441	98
Na^+^/Ca^+^/K^+^ and other cation porters, antyporters and symporters	Bacteroidetes	A	2	30	69
		E	4	666	83
TonB, SusD transport proteins	Bacteroidetes	A	16	1333	91
		E	9	7191	83
	Clostridiales	H	1	1404	94
**GENERAL METABOLISM**					
Formate dehydrogenases	Bacteroidetes	A	2	20	60
		E	1	59	61
Glycine dehydrogenase	Bacteroidetes	A	1	36	71
		E	1	213	98
ATP synthase and ATPase	Bacteroidetes	A	9	101	68
		E	5	389	70
Glucose metabolism	Bacteroidetes	A	1	12	52
		E	3	717	88
GTPases	Bacteroidetes	A	3	42	75
		E	4	705	80
NAD/NADH metabolism	Bacteroidetes	A	12	516	87
		E	8	1514	86
Peptidases/Oligopeptidases	Bacteroidetes	A	14	274	72
		E	9	1676	88
	Clostridiales	H	1	7350	100
Pyruvate metabolism	Bacteroidetes	A	10	439	96
		E	4	1626	84
	Clostridiales	B	1	208	62
Ribosomal metabolism	Bacteroidetes	A	11	215	79
		E	5	1655	83
Gliding and motility	Bacteroidetes	A	3	208	80
		E	1	44	78
Glycoside hydrolase	Bacteroidetes	E	6	791	90
Glycosyl transferases	Bacteroidetes	E	5	392	75
**DNA/RNA METABOLISM**					
DNA metabolism	Bacteroidetes	A	13	216	73
		E	18	4860	92
RNA metabolism	Bacteroidetes	A	2	47	70
		E	3	985	96
	Clostridiales	B	1	203	61
Transcription related proteins	Bacteroidetes	A	8	261	85
		E	2	927	100
	Clostridiales	B	1	248	52

*Selected CDS with annotated function and mapped mRNA transcripts from bins A, B, E, and H*.

*Only those CDS where the mRNA transcripts covered more than 50% of the CDS were used (See Materials and Methods for details)*.

**Figure 8:**
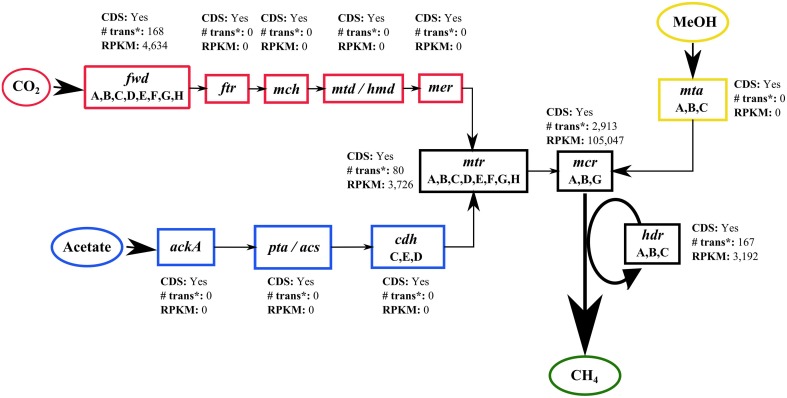
**Active methanogenic pathways**. Schematic representation of the specific enzymes of the three methanogenic pathways, hydrogenotrophic (CO_2_), acetoclastic (Acetate) and methylotrophic (MeOH) based on the KEGG pathway modules M00567, M00357, and M00356. For each enzyme, the presence of CDS, the total number of mRNA transcripts mapped and the total RPKM value is shown. trans, mRNA transcripts; MeOH, methanol. Enzyme abbreviations: *fwd*, formylmethanofuran dehydrogenase; *ftr*, formylmethanofuran–tetrahydromethanopterin N-formyltransferase; *mch*, methenyltetrahydromethanopterin cyclohydrolase; *mtd*, methylenetetrahydromethanopterin dehydrogenase; *hmd*, 5,10-methenyltetrahydromethanopterin hydrogenase; *mer*, 5,10-methylenetetrahydromethanopterin reductase; *mtr*, tetrahydromethanopterin S-methyltransferase; *mcr*, methyl-coenzyme M reductase; *hdr*, heterodisulfide reductase; ackA, acetate kinase; *pta*, phosphate acetyltransferase; *acs*, acetyl-CoA synthetase; *cdh*, acetyl-CoA decarbonylase/synthase complex: *mta*, methyl-Co(III) methanol-specific corrinoid protein.

Using GenDB, it was not possible to automatically assign a specific function to the CDS detected in bin F, *Methanocalculus*. However, when the identified CDS were blasted against a database containing the enzymes from the three different methanogenesis pathways, key enzymes of these pathways were detected and their activity could be assessed using the RPKM value calculated with ReadXplorer (Figure 8). Coding sequences from enzymes from all three pathways were detected in bin F. However, only a few enzymes appeared to be active. None of the specific enzymes of the methylotrophic and the acetoclastic pathways recruited mRNA transcripts, whereas the enzyme formylmethanofuran dehydrogenase (*fwd*) specific of the hydrogenotrophic pathway, recruited 168 mRNA transcripts with an RPKM value of 4,634 (Figure 8). The common enzymes of the three pathways were all actively transcribed. Among these, the most active enzyme was methyl-coenzyme M reductase (*mcr*), which catalyzes the last step of the methanogenesis (Friedrich, 2005), with an RPKM value of 105,047 (Figure 8).

## Discussion

In the work presented here we show that it is possible to use a haloalkaline anaerobic microbial community for the anaerobic digestion of the microalgae *Spirulina* at alkaline conditions (pH 10 and 2.0 M Na^+^), and that the obtained biogas is rich in methane. The study of the haloalkaline anaerobic community indicates that Bacteroidetes and *Methanocalculus* play a key role in the anaerobic digestion at these extreme conditions.

### Biogas rich in methane

As expected, by applying alkaline conditions in our anaerobic digester, methane rich biogas was obtained (Figure 1). This was due to the fact that the solubility of carbon dioxide in a solution is determined mainly by the pH of the solution and its buffering capacity. Because of the high pH, the (bi)carbonate concentration in the medium can be high while a driving force for carbon dioxide absorption is maintained. Since all absorbed/hydrated carbon dioxide immediately reacts with OH^−^ to form (bi)carbonate, CO_2_ absorption kinetics are faster than at neutral pH. With the high pH and alkalinity used in our experiments, the reactor's medium acted as a CO_2_ scrubber and the carbon dioxide remained in solution as carbonates (alkalinity) which resulted in a low percentage of CO_2_ in the headspace (Figure 1). In all three experiments, this scrubber effect produced biogas with a high percentage of methane that ranged from 77 to 88% (Table 3) with peaks up to 96% in the Alk-HRT (Figure 1A), 92% in the Alk-OLR (Figure 1B) and 90% in the Alk-Opt (Figure 1C). These values for methane content are higher than the 78% obtained in the study of Van Leerdam et al. (2008), which is, to date, the only other study of anaerobic digestion at high pH. A further interesting and important observation was that, as in the case of van Leerdam et al., no H_2_S was detected in the biogas during all three experiments. This high methane content and the absence of H_2_S make this biogas suitable to be used as biomethane for vehicles and national gas supply grids with none or only a minor upgrade. For example, in Germany the minimum required content of methane in biomethane is 96%, in Norway 95% and in Netherlands 88% (Persson et al., 2006).

### Biogas production

Daily biogas production ranged from 35 to 60 ml per day in all three reactors Alk-HRT, Alk-OLR, and Alk-Opt (Figure 1), comparable to what was found previously with methanethiol as substrate which, to date, is the only other known substrate digested at alkaline conditions (Van Leerdam et al., 2008). This daily biogas production is, however, low if compared to studies performed at mesophilic pH and alkalinity using *Spirulina* as substrate. Samson and Leduy (Samson and LeDuy, 1982, 1983, 1986) obtained between 260 and 350 ml of methane from the continuous anaerobic digestion of *Spirulina* while Varel et al. (1988) obtained between 300 and 470 ml of methane.

### Identification of optimal HRT and OLR and key factors

As this is the first study of anaerobic digestion of complex OM using a haloalkaline microbial community operated at extreme alkaline conditions it was necessary to identify the basic optimal process parameters, HRT and OLR, in order to maximize the biogas production.

The selection of the different residence times in the Alk-HRT, and the actual duration of each period, was adapted to the observed circumstances in order to avoid reactor failure at each given time point. The choice of HRTs was also determined by the type of substrate used, *Spirulina*, a protein rich microalga (60–75% of proteins dry weight) (Ortega-Calvo et al., 1993). The anaerobic digestion of protein rich substrates generates high amounts of nitrogen in the form of ammonium (NH_4_) which, at normal pH conditions, does not affect the biogas production, unless a high OLR is applied (Sialve et al., 2009). However, at high pH, and according to the equation by Anthonisen et al. (1976), the released ammonium is present in the medium mainly in the dissociated form NH_3_ (Figure 2A), a form which is highly toxic for methanogenic archaea and its accumulation can cause reactor failure (Sterling et al., 2001; Strik et al., 2006). This characteristic influenced the selection of the different HRTs tested and it clearly affected the biogas production.

Initially, 20 days HRT was used (P-I) adopted from own experiments of anaerobic digestion of *Spirulina* at mesophilic pH conditions. However, at alkaline conditions, with 20 days HRT a rapid accumulation of NH_3_ occurred (Figure 2A). To avoid eventual reactor failure, it was decided to drastically reduce the HRT from 20 to 5 days (P-II). This, as expected, markedly reduced the levels of NH_3_, but at the same time it caused a reduction in the biogas production. As the levels of NH_3_ were low in this period, the sudden drop in the daily biogas production was mainly attributed to a washout of the active biomass, which occurs when microorganisms are purged in excess from the anaerobic reactor causing a reduction of the biogas production (Zhang and Noike, 1994; Ward et al., 2014). Reducing the HRT implied an increase in the amount of sludge exchanged daily, in this case from 75 to 300 ml (Table 2), therefore excessive purging of biomass occurred. The high biomass loss became apparent by a reduction in the levels of OM measured as total COD which was reduced from 20 to 13 g O_2_ L^−1^. At this point, and to reduce the washout, the HRT was increased to 10 days (P-III). However, in this period the biogas production remained the same and the washout continued. The cause of this high loss of biomass in P-II and P-III could be mainly attributed to the low presence of granules or aggregates in the alkaline reactor. A microscopic observation of sludge samples from these two periods revealed an almost complete absence of granules or aggregates (Supplementary Figure 1). The formation of microbial aggregates contributes to (i) the precipitation of the microbial biomass which reduces its washout and (ii) to the interaction between the different microorganisms which contributes to a higher biogas production (Yu et al., 2001; Borja, 2011). Thus, biomass washout due to a possible low formation of aggregates resulted in a decrease of biogas production when compared to the previous period (Figure 2).

In order to enrich the methanogenic microbial community, the HRT was increased to 30 days (P-IV). Increasing the time of residence implies a reduction in the medium exchange, in this case from 150 to 50 ml (Table 2), which can lead to an accumulation of inhibitory substances. As can be seen in Figure 2, increasing the HRT to 30 days initially stimulated the biogas production. Eventually, the daily biogas production was affected, marked by sudden drops on days 99 and 115. These drops were attributed mainly to an inhibitory effect due to accumulation of VFAs, OM and especially free NH_3_ which led to an almost complete failure of the Alk-HRT reactor (Figure 2). To recover the reactor, it was necessary to reduce the accumulated inhibitory compounds by applying a 5 day period of exchanging 50 ml of sludge with fresh alkaline medium without *Spirulina*. After these 5 days the levels of inhibitory substances were drastically reduced (Figure 2). The HRT was then set to 15 days and the feeding resumed. Fifteen days HRT was chosen in order to reach a compromise between avoiding accumulation of inhibitory substances and excess washout of biomass. For 15 days HRT, the daily biogas production was the highest achieved during this experiment, 50 ml biogas day^−1^, (Table 3) the NH_3_ was stable (between 900 and 1,000 mg L^−1^) and the biogas production was constant for 100 days (Figure 2). From the different hydraulic retention times studied and considering all observed factors it was concluded that the optimal HRT for the anaerobic digestion of *Spirulina* at alkaline conditions with an OLR of 1.0 g L^−1^ day^−1^ (dry weight), was 15 days. At this HRT, the highest SBP was obtained, 37 ml g VS^−1^, and the highest percentage of *Spirulina* converted to methane, measured as percentage of biodegradability (Raposo et al., 2011), 5%, was achieved (Table 3). For this HRT, both accumulation of inhibitory substances and biomass washout were kept at levels that favored biogas production.

Daily biogas production was also affected by the amount of OM fed to the reactor and whenever the OLR was increased the biogas production also increased (Alk-OLR experiment) (Figure 1B). However, the increase in biogas production did not correlate linearly with the increase in the OLR. In Alk-OLR, from P-I to P-II the OLR was doubled, from 0.25 to 0.5 g *Spirulina* L^−1^ day^−1^, while the biogas production increased by 77%. Similarly, from P-II to P-III, the OLR was again doubled, yet the biogas production only increased by 26%. These results indicate that the additional substrate provided was not effectively converted to methane. Instead, it accumulated mainly as total and soluble OM (increasing COD values) (Figure 3). This accumulation eventually caused a slow but constant reduction of the daily biogas production during P-III which was attributed to a substrate overload which generally occurs when the microbial community is unable to completely digest the supplied substrate and toxic compounds accumulate (Salminen and Rintala, 2002; González-Fernández and García-Encina, 2009; Kwietniewska and Tys, 2014). Efforts were made to try to recover the anaerobic reactor but were unsuccessful.

From the three different OLR tested, 0.25 g L^−1^ day^−1^ was determined to be the optimal. With this OLR, substrate overload was avoided and no accumulation of OM and VFAs occurred and, in combination with 15 days HRT, accumulation of free NH_3_ was also prevented. Additionally, the highest daily biogas production was achieved, as well as the highest SBP per gram of VS added, 56 ml. Moreover, with this OLR the biodegradability of *Spirulina* was also the highest obtained, 7% (Table 3). The low OLR applied in combination with the 15 days HRT thus avoided both, accumulation of toxic compounds and bacterial washout.

### Biogas production at optimal HRT and OLR

With the selected optimal parameters, 15 days HRT and 0.25 g *Spirulina* L^−1^ day^−1^ OLR, the biogas production was constant for a period of 67 days (Figure 4). Performing the digestion at the optimal conditions avoided the problems encountered during the previous experiments. NH_3_ was kept below the inhibitory threshold, 150–900 mg L^−1^ (Angelidaki and Ahring, 1993; Calli et al., 2005), and volatile fatty acids and OM also remained controlled (Figure 4). The constant levels of both total organic matter (COD_T_) and soluble organic matter (COD_*S*_) and the controlled levels of acetic acid indicated that the *Spirulina* degradation rate and its conversion to methane were in equilibrium. In addition, the low NH_3_ levels in the reactor's medium avoided the inhibition of methanogens (Figure 4). These results indicate that, at the set conditions (15 days HRT and 0.25 g *Spirulina* L^−1^ day^−1^ OLR), the microbial community was able to successfully digest part of the supplied substrate and was not affected by inhibitory substances. This resulted in the highest daily biogas production and SBP obtained in our experiments (Table 3). At these specific settings also the highest percentage of BD_CH4_ of all three experiments was achieved, 11%. This increase in the SBP and BD_CH4_ was attributed to several factors: (i) a better equilibrium between the hydrolysis and the consumption rates which avoided accumulation of toxic compounds that could cause inhibition of the anaerobic community; (ii) reduced washout of the active biomass combined with the removal of sufficient inhibitory substances and (iii) the inoculum used for the Alk-Opt experiment was a relatively fresh inoculum which possibly contained a more active microbial community compared to the one used in the Alk-HRT and Alk-OLR experiments.

### Identification of key players by the taxonomic and functional analysis of the haloalkaline microbial community

Metagenomics in combination with metatranscriptomics are two approaches to elucidate the composition and the functional activity of complex microbial communities (Gilbert et al., 2008; Thomas et al., 2012). In the present study, binning of assembled contigs into provisional whole genome sequences of abundant community members, combined with mapping of transcriptome reads, provided firsthand information about the ecological function of each of these organisms.

#### Hydrolysis and uptake of substrate

At mesophilic pH, the first three steps of anaerobic digestion, hydrolysis, acidogenesis and acetogenesis, can be performed by bacteria of the phyla Firmicutes, Bacteroidetes, and Thermotoga (Al Seadi et al., 2008; Schlüter et al., 2008; Jaenicke et al., 2011; Pavlostathis, 2011). In the case of the anaerobic digestion at alkaline conditions these groups of bacteria also appeared to be responsible for these processes but in different proportions than previously found (Table 4).

Bacteroidetes, closely related to the uncultured Bacteroidetes “ML635J-40 aquatic group” appeared to be the dominant bacteria present in the alkaline reactor (bins A and E). These Bacteroidetes, part of the Cytophaga–Flavobacterium–Bacteroides group (CFB), have been previously identified in soda lakes and alkaline environments such as the Mono lake (Humayoun et al., 2003), lake Magadi (Baumgarte, 2003), and the Lonar crater lake (Wani et al., 2006). Phylogenetically, the 16S rRNA sequences identified in the two Bacteroidetes bins, were closely related to sequences cloned from alkaline environments (Figure 5). The CFB group is an important group of bacteria present in aquatic and haloalkaline environments which participate in the degradation of OM and complex polysaccharides (Humayoun et al., 2003; Brettar et al., 2004; Rees et al., 2004). Some of their members are able to utilize proteins as substrate (Chen and Dong, 2005) and all the identified organisms can grow in the presence of salt (Denger et al., 2002; Brettar et al., 2004; Na et al., 2009). The function of “ML635J-40 aquatic group” regarding anaerobic digestion is not fully understood, as to date no pure cultures have been obtained. In the alkaline anaerobic reactor they seem to play an important role in the breakdown of substrate, *Spirulina* algae. As can be seen in Table 5, they possess a broad range of active membrane transport proteins such as ABC transporters, oligopeptide and amino acid transporters and several TonB, SusD and related transport systems. ABC transporters are a group of broad spectrum substrate uptake proteins involved in the uptake of peptides, sugars, and other nutrients (Davidson et al., 2008). TonB is a multiple purpose transport system involved in the uptake of carbohydrates and vitamins (Schauer et al., 2008; Noinaj et al., 2010) while SusD belongs to a series of four outer membrane proteins involved in the binding and degradation of starch (Shipman et al., 2000; Mackenzie et al., 2012). The high RPKM values obtained for these proteins in both “ML635J-40 aquatic group” bins, A and E, compared to the Clostridiales bins, B and H (Table 5) suggested that they are the main bacteria responsible for the degradation and uptake of peptides and other macromolecules.

It is not a surprise that, as most of the identified bacteria in the alkaline anaerobic reactor were related to known halophiles and alkaliphiles (Table 4), multiple enzymes involved in the adaptation strategies of halotolerant and haloalkaline bacteria were identified among the transcribed CDS (Table 5). It is important to highlight the detection of several Na^+^ and K^+^ porters and antiporters which are commonly used to allow flow of these ions through the bacterial membrane in order to maintain the intracellular pH at mesophilic conditions, one of the two strategies of halophiles and alkaliphiles to cope with the osmotic pressure (Mesbah et al., 2007; Kivistö and Karp, 2011). It is also worth noting that among the active transport systems of the “ML635J-40 aquatic group” we could identify several extremely active glycine/betaine/choline transporters (Table 5). These enzymes participate in the uptake of osmoprotectants and compatible solutes, and are present in halotolerant bacteria which accumulate them in the form of glycine, betaine, ectoine, and choline in order to cope with the high salt concentrations present in the alkaline medium (Ventosa, 2006; Ma et al., 2010; Mesbah and Wiegel, 2012).

Clostridiales (bins B and H) appeared to play an important role in the anaerobic digestion at alkaline conditions. The 16S rRNA sequences identified among these two bins were phylogenetically related to several clones and Clostridiales such as *Proteinivorax, C. elmenteitii, Anaerobranca*, and *Tindallia* isolated from alkaline environments (Figure 6). These bacteria are known to be able to grow at high pH, some require sodium and can utilize yeast extract as carbon and energy source (Jones et al., 1998; Yumoto, 2002; Kevbrin et al., 2013; Yutin and Galperin, 2013). The functional characterization of these two bins indicated the presence of substrate transporters, but in contrast to the “ML635J-40 aquatic group,” their activity was lower (Table 5). They contain, however, oligopeptide/dipeptide transport proteins with high RPKM values, which is in accordance to the fact that they can use proteins and yeast extract as nutrients (Engle et al., 1995; Kevbrin et al., 1998, 2013), and bin B also expresses an active substrate binding protein (Table 5). These results suggest, that, as in the case of the anaerobic digestion process at neutral pH, members of the Clostridiales are involved in the degradation of OM (Schlüter et al., 2008; Jaenicke et al., 2011; Kovács et al., 2013; Li et al., 2013).

Multiple CDS encoding enzymes from general metabolic and DNA/RNA processes were also transcribed in the Bacteroidetes and Clostridiales bins (Table 5). Transcripts of multiple peptidases and oligopeptidases were identified in bins A, E, and H. For example, bin E contained nine active peptidases such as peptidases M23, M28, and S9 families among others while bin H contained one extremely active peptidase with a RPKM value of 7,350. Several ATP synthases, GTPases and related enzymes were also active in both Bacteroidetes bins. Enzymes from the pyruvate metabolism like pyruvate kinases and pyruvate carboxylases were also transcribed in bins A and B and especially in bin E, which contained four pyruvate metabolism CDS with mRNA transcripts mapped to an RPKM of 1,626 (Table 5). The two bins assigned to “ML635J-40 aquatic group,” bins A and E also contained several formate and glycine dehydrogenases, enzymes that catalyze the oxidation of formate to CO_2_ and of glycine to NH_3_, both metabolites present in relatively high abundance in the alkaline medium. CDS encoding enzymes related to DNA and RNA metabolism were also transcribed in bins A, B, and E (Table 5). It is worth noting the presence of 18 DNA metabolism related CDS, such as DNA polymerase III and DNA ligase, in bin E which were highly transcribed. This, in addition to the three RNA related enzymes which were also highly transcribed, suggests that this particular population was growing actively. According to the results from Table 5 it appears that, in accordance to their abundance (Table 4), the Bacteroidetes of the “ML635J-40 aquatic group” played the most important role in the degradation and transformation of OM in the alkaline anaerobic reactor. Additionally, the detection of transcripts of these general metabolic enzymes in combination to the detection of the multiple transport proteins indicate that the hydrolytic bacteria present in the alkaline reactor were actively hydrolyzing the supplied substrate.

Other halotolerant bacteria were also identified in the alkaline reactor. For example, bins C, D, and G were assigned to Firmicutes of the Halanaerobiales order (Table 4). The 16S sequences present in these bins were phylogenetically closely related to two uncultured clones and to other Halanaerobiales such as *Orenia* and *Halanaerobium*, all identified or isolated in soda lakes or hypersaline environments (Figure 6). *Orenia, Halanaerobium*, and related Halanaerobiales have been identified in multiple soda lakes, such as lake Magadi and Great Salt Lake. They are known to be moderately halophilic and they are able to ferment glucose, fructose and pectin among other substrates to produce acetate, CO_2_ and H_2_ (Baumgarte, 2003; De la Haba et al., 2011; Kivistö and Karp, 2011). In addition to these Halanaerobiales, an unbinned 16S rRNA sequence closely related to *Natronovirga wadinatrunensis*, a member of the Natranaerobiales order, was also identified among the contigs from assembly A. Assembly B also yielded several 16S sequences which were assigned to the Natranaerobiales and Halanaerobiales clades (Figure 6). The function of this group of organisms is still unclear as no function was automatically assigned to the detected CDS via GenDB. However, these bacteria are active as mRNA transcripts were mapped to a high percentage of the identified CDS (Supplementary Table 3). A manual annotation of several of these active CDS showed the presence of multiple ABC transporters, extracellular solute binding proteins, ATP dependent proteases and peptidases (data not shown) indicating that these groups of bacteria were also actively contributing to the degradation of *Spirulina*.

#### Methanogenesis

The final step in the anaerobic digestion process is the production of methane carried out by methanogenic archaea. Methanogenesis in alkaline environments has been widely studied for the last decades (Oremland et al., 1982; Zhilina and Zavarzin, 1994; Sorokin and Kuenen, 2005a; Grant, 2006; McGenity, 2010) but so far only one study by Van Leerdam et al. (2008) described a methanogenic population in lab-scale anaerobic alkaline reactors. In that study, *Methanolobus oregonensis*, a methylotrophic member of the Methanosarcinales, was the dominant methanogen. In recent studies, amplification of the *mcrA* marker gene indicated the presence of Methanobacteriales and Methanomicrobiales, the latter closely related to the genus *Methanocalculus*, in soda lake sediments originating from the same lake as the inoculum used for the alkaline reactor (Nolla-Ardèvol et al., 2012; Sorokin et al., 2015).

In the alkaline anaerobic reactor, a single population of methanogens, possibly *Methanocalculus*, dominated among the archaeal community (Table 4). Bin F contained one 16S rRNA sequence which was classified as *Methanocalculus* (Supplementary Table 2). Phylogenetically, this sequence was closely related to *Methanocalculus* sp. *AMF-Bu2* (98% similarity at DNA level), identified in sediments from soda lakes of the Kulunda Steppe (Altai, Russia), the same lake system from which the inoculum for the alkaline reactor was obtained, and to *Methanocalculus natronophilus* (98% similarity at DNA level), isolated from sediments of soda lakes of the Tanatar II system, also in the Kulunda region (Zhilina et al., 2013) (Figure 7). As expected, the 16S sequence of the identified methanogen clustered together with 16S sequences of other halotolerant Methanomicrobiales and it was clearly distant from Methanomicrobiales isolated and identified in neutral pH anaerobic reactors. Members of the genus *Methanocalculus*, which are known to be hydrogenotrophic (Ollivier et al., 1997), have been identified in other alkaline environments such as Lonar crater lake in India (Antony et al., 2012), Ethiopian soda lakes (Lanzén et al., 2013), and a hypersaline oil reservoir (Ollivier et al., 1997). All known members of this genus, including *M. halotolerans* (97% similarity at DNA level with contig01776), have restricted pH growth ranges from 6.7 to 8.3 (Ollivier et al., 1997; Lai et al., 2002), with one exception, *Methanocalculus natronophilus. M. natronophilus* is the so far only known strictly alkaliphilic member of this genus, it can only grow at pH between 8.0 and 10.2, with an optimum between pH 9.0 and 9.5, it also requires between 0.5 and 1.6 M of carbonates and from 0.9 to 3.3 M of Na^+^. *M. natronophilus* is a hydrogenotrophic methanogen and cannot use acetate as substrate for methanogenesis albeit it requires it for growth as carbon source (Zhilina et al., 2013).

When the detected CDS from bin F were blasted against a database containing all enzymes from the three different methanogenesis pathways, multiple enzymes could be detected (Figure 8). Despite the fact that some reports suggest that in alkaline and hypersaline environments methane is also formed from methylated compounds such as methanol, dimethylsulfide and methylated amines through the methylotrophic pathway (Jones et al., 1998; McGenity, 2010), in the alkaline anaerobic reactor, this pathway appeared to be less active as no mRNA transcripts mapping the *mta* enzyme were detected (Figure 8). As suggested by the results from our previous work (Nolla-Ardèvol et al., 2012) and the absence of 16S rRNA sequences belonging to acetoclastic methanogens among the two assemblies, the acetoclastic pathway also appeared to be inactive as no mRNA transcripts were mapped to the CDS of the specific enzymes from this pathway. The low or absent activity of the acetoclastic pathway could contribute to explain why during the operation of the different alkaline reactors, the levels of acetic acid were relatively high (Figures 2B, 4). It is possible that in our alkaline reactors, acetate was not used as substrate for methanogenesis. With our current knowledge of the microbial community, the fate of this compound is still unclear. For one of the specific enzymes of the hydrogenotrophic pathway, however, mRNA transcripts were detected (Figure 8). The presence of transcripts that mapped to formylmethanofuran dehydrogenase (*fwd*), an enzyme that catalyzes the first step in the hydrogenotrophic pathway (Karrasch et al., 1989), in addition to the detection of an active tetrahydromethanopterin S-methyltransferase (*mtr*), suggest that the hydrogenotrophic pathway was the most productive methanogenesis pathway. This is in accordance with the finding that the dominant organism of the methanogenic population is very likely *Methanocalculus* which is a hydrogenotrophic methanogen able to use CO_2_ and formate for the production of methane (Zhilina et al., 2013). The two enzymes common to the three pathways, methyl coenzyme-M reductase (*mcr*) and heterodisulfide reductase (*hdr*), both recruited a high number of transcripts with a high RPKM value indicating that the methanogenic population was actively producing methane.

The functional analysis of bin F also revealed the presences of an active formate transporter and a glycine/betaine ABC transporter substrate-binding protein, among the top 10 most active CDS. The glycine/betaine transporters possibly play a similar role as the ones detected among bins A and E, and contribute to maintain the intracellular osmotic pressure. The activity of formate transporters such as FocA, responsible for the uptake of formate in anaerobic cells (Suppmann and Sawers, 1994) suggests that in the alkaline reactor *Methanocalculus* not only used CO_2_ and H_2_ as substrates for methane production, but as already stated by Zhilina et al. (2013) and Sorokin et al. (2015) it also used formate.

#### Microbial structure of the anaerobic digestion process at alkaline conditions

The microbial community of the anaerobic digestion process at neutral pH is generally dominated by Clostridiales, with abundances from 30 to 80%, Bacteroidetes and Bacilli (10–15%) and, in minor percentages, Proteobacteria, Actinobacteria, Sphingobacteriia, and Thermotogae among many others, with methanogens representing between 3 and 10% of the total community (Schlüter et al., 2008; Rivière et al., 2009; Wirth et al., 2012; Li et al., 2013; Sundberg et al., 2013; Ziganshin et al., 2013). At alkaline conditions, however, significant differences were observed, not only with regard to the type of bacteria present, mostly halotolerant and alkaliphilic, but also to their relative abundances (Table 4). The most remarkable difference was the high abundance of Bacteroidetes (27%) and Halanaerobiales (~15%), which appeared to be responsible for the main breakdown of the organic matter, and fewer Clostridiales (13%). Also, with regards to the methanogenic community, clear differences were observed regarding the involved taxa when compared to neutral pH reactors. In our alkaline system, *Methanocalculus*, an alkaliphilic hydrogenotrophic methanogen, clearly dominated over the rest of methanogenic archaea.

The results presented here suggest that at the tested alkaline conditions, pH 10 and 2.0 M Na^+^, the microbial community responsible for the anaerobic digestion of OM differs from neutral pH anaerobic microbial communities, not only regarding the fact that the identified bacteria and archaea are mostly halotolerant and alkaliphilic, but also with regard to the relative abundance of the major players.

## Conclusions and perspectives

In this work we have shown that it is possible to use a haloalkaline anaerobic microbial community for the production of biogas. The anaerobic digestion of the microalga *Spirulina* was possible at alkaline conditions, pH 10, 2.0 M Na^+^. Continuous biogas production was observed and the obtained biogas was rich in methane (up to 96%). However, the biogas production was low and affected by several factors such as NH_3_-N and volatile fatty acids accumulation. These drawbacks might be overcome by using alternative substrates and/or reactor configurations which allow the combination of long biomass retention time and a short HRT (e.g., membrane reactors). The haloalkaline microbial community present in the alkaline reactors is dominated by Bacteroidetes, Halanaerobiales and other halotolerant and alkaliphilic bacteria which are actively involved in the degradation of organic matter. *Methanocalculus* clearly dominates the methanogenic population while the hydrogenotrophic pathway appears to be the most active pathway for the production of methane at such alkaline conditions.

Anaerobic digestion at alkaline conditions could also be beneficial for the treatment of wastewaters that today are difficult to process such as waste waters from the fishery industry which contain high levels of Na^+^ (Sandberg and Ahring, 1992; Chowdhury et al., 2010) and alkaline wastewaters such as waste streams from brewery industries, concentrated sugar wastewaters and leather tannery wastewaters among many others (Rosenwinkel et al., 2005; Lofrano et al., 2013). Moreover, anaerobic alkaline digestion could also be used to produce biogas from wheat straw and similar lignocellulosic substrates in one single pot reaction as the alkaline medium could act as a pretreatment making the hydrolysis of these substrates more efficient.

## Author contributions

VN designed and performed the experiments presented herein, evaluated the data and drafted the manuscript. MS conceived the study, assisted in experimental design and in drafting the manuscript. HT supervised the work, assisted in evaluation of the data and drafted the manuscript. All authors participated in the experimental design, evaluation of the data, read and approved the final manuscript.

### Conflict of interest statement

The authors declare that the research was conducted in the absence of any commercial or financial relationships that could be construed as a potential conflict of interest.

## Abbreviations

HRT: Hydraulic retention time
OLR: Organic loading rate
OM: Organic matter
COD_T_: Total chemical oxygen demand
COD_S_: Soluble chemical oxygen demand
VFAs: Volatile fatty acids
SMP: Specific methane potential
BD_CH4_: percentage of substrate conversion to methane
Alk-HRT: Alkaline HRT reactor
Alk-OLR: Alkaline OLR reactor
Alk-Opt: Alkaline Optimal reactor
CDS: Coding DNA Sequence
CFB: Cytophaga-Flavobacteria-Bacteroides group.
