# Supplementary material for: Anaerobic digestion of the microalga Spirulina at extreme alkaline conditions: biogas production, metagenome, and metatranscriptome
Source: Front Microbiol. 2015 Jun 22;6:597. doi: 10.3389/fmicb.2015.00597 (PMC4475827; doi:10.3389/fmicb.2015.00597)
Supplement: Supplementary file 1 [file Table1.PDF]

Supplementary Table-1

Anaerobic digestion of the microalga *Spirulina* at extreme alkaline conditions: biogas production, metagenome and metatranscriptome

Vimac Nolla-Ardèvol[1*], Marc Strous[1,2,3], Halina E. Tegetmeyer [1,3,4]

[1]Institute for Genome Research and Systems Biology, Center for Biotechnology, Bielefeld University, Bielefeld, Germany.

[2]Department of Geoscience, University of Calgary, Calgary, AB, Canada.

[3]Microbial Fitness Group, Max Planck Institute for Marine Microbiology, Bremen, Germany.

[4] HGF-MPG Group for Deep Sea Ecology and Technology, Alfred Wegener Institute, Helmholtz Centre for Polar and Marine Research, Bremerhaven, Germany

Suppl. Table 1. DNA Sequencing statistics

Alkaline metagenome sequencing data and assemblies statistics

Sequencing data	Bases	Reads	Mean Read length	GC %
PGM raw data	952 MB	4,602,427	237	46
Post Trimmomatic and quality trimming	428 MB	2,032,005	274	48

Assembled contigs

Assemblies	# Submitted reads	Minimum read length	# Contigs	# Contigs > 500bp	N50 contig size	Mean contig size	Largest contig size
Assembly A*	2,032,005	100 (bp)	31,258	14,151	1,714	1,358	111,125
Assembly B*	2,032,005	100 (bp)	144,389	29,864	1,084	1,051	59,130

* See Material and Methods for details about assembly settings.
