# Supplementary material for: Anaerobic digestion of the microalga Spirulina at extreme alkaline conditions: biogas production, metagenome, and metatranscriptome
Source: Front Microbiol. 2015 Jun 22;6:597. doi: 10.3389/fmicb.2015.00597 (PMC4475827; doi:10.3389/fmicb.2015.00597)
Supplement: Supplementary file 2 [file Table2.PDF]

Supplementary Table-2

Anaerobic digestion of the microalga *Spirulina* at extreme alkaline conditions: biogas production, metagenome and metatranscriptome

Vimac Nolla-Ardèvol[1*], Marc Strous[1,2,3], Halina E. Tegetmeyer [1,3,4]

[1]Institute for Genome Research and Systems Biology, Center for Biotechnology, Bielefeld University, Bielefeld, Germany.

[2]Department of Geoscience, University of Calgary, Calgary, AB, Canada.

[3]Microbial Fitness Group, Max Planck Institute for Marine Microbiology, Bremen, Germany.

[4] HGF-MPG Group for Deep Sea Ecology and Technology, Alfred Wegener Institute, Helmholtz Centre for Polar and Marine Research, Bremerhaven, Germany

Suppl. Table 2. Contigs containing 16S rDNA sequences of the 9 selected bins

16S rDNA taxonomical assignment of binned and unbinned contigs from assembly A and contigs from assembly B performed with the SILVA and RDP databases. Assignment was done with 80% minimum identity. Numbers in brackets indicate the highest percentage of identity. No number indicates 100% identity.

Ass*	Bin	Contig	DB	Phylum	Class	Order	Family	Genus
A	A	Contig08670	SILVA	Bacteroidetes	Bacteroidia	Bacteroidales	ML635J-40 aquatic group	
			RDP	Bacteroidetes	Bacteroidia (61)			
		Contig11253	SILVA	Bacteroidetes	Bacteroidia	Bacteroidales	ML635J-40 aquatic group	
			RDP	Bacteroidetes	Flavobacteria (63)			
		Contig24233	SILVA	Bacteroidetes	Bacteroidia	Bacteroidales	ML635J-40 aquatic group	
			RDP	Bacteroidetes (99)	Incertae sedis (24)			
A	B	Contig02281	SILVA	Firmicutes	Clostridia	Clostridiales		
			RDP	Firmicutes	Clostridia	Clostridiales	Clostridiaceae (74)	
A	C	Contig01919	SILVA	Firmicutes	Clostridia	Halanaerobiales	Halobacteroidaceae	
			RDP	Firmicutes	Clostridia	Halanaerobiales	Halobacteroidaceae	*Orenia (76)*
A	D	Contig03844	SILVA	Firmicutes	Clostridia	Halanaerobiales	Halanaerobiaceae	*Halanaerobium*
			RDP	Firmicutes	Clostridia	Halanaerobiales (98)	Halanaerobiaceae (91)	*Halocella (52)*
		Contig21218	SILVA	Firmicutes	Clostridia	Halanaerobiales	Halanaerobiaceae	
			RDP	Firmicutes	Clostridia	Halanaerobiales (99)	Halanaerobiaceae (98)	*Halothermothrix (64)*
		Contig25077	SILVA	Firmicutes	Clostridia	Halanaerobiales	Halanaerobiaceae	*Halanaerobium*
			RDP	Firmicutes	Clostridia	Halanaerobiales	Halanaerobiaceae (98)	*Halanaerobium (61)*
A	E	Contig05178	SILVA	Bacteroidetes	Bacteroidia	Bacteroidales	ML635J-40 aquatic group	
			RDP	Bacteroidetes	Flavobacteria (55)			
		Contig25151	SILVA	Bacteroidetes	Bacteroidia	Bacteroidales	ML635J-40 aquatic group	
			RDP	Bacteroidetes	Flavobacteria (38)			
		Contig26930	SILVA	Bacteroidetes	Bacteroidia	Bacteroidales	ML635J-40 aquatic group	
			RDP	Bacteroidetes (98)	Sphingobacteria (31)			
		Contig29659	SILVA	Bacteroidetes	Bacteroidia	Bacteroidales	ML635J-40 aquatic group	
			RDP	Bacteroidetes (91)	Flavobacteria (71)			
A	F	Contig01776	SILVA	Euryarchaeota	Methanomicrobia	Methanomicrobiales	Incertae Sedis	*Methanocalculus*
			RDP	Euryarchaeota	Methanomicrobia	Methanomicrobiales	Incertae Sedis	*Methanocalculus*

* Ass: Assembly used. See Material and Methods section for details

Suppl. Table 2. Continuation

Ass*	Bin	Contig	DB	Phylum	Class	Order	Family	Genus
A	G	Contig11185	SILVA	Firmicutes	Bacilli	Bacillales	Bacillaceae	
			RDP	Firmicutes (97)	Clostridia (86)	Halanaerobiales (61)		
		Contig12844	SILVA	Firmicutes	Clostridia	Halanaerobiales	Halanaerobiaceae	*Halanaerobium*
			RDP	Firmicutes	Clostridia	Halanaerobiales	Halanaerobiaceae	*Halanaerobium*
		Contig14076	SILVA	Firmicutes	Clostridia			
			RDP	Firmicutes (89)	Clostridia (73)			
		Contig18213	SILVA	Firmicutes				
			RDP	Firmicutes (68)				
		Contig18995	SILVA	Firmicutes	Clostridia	Halanaerobiales	Halanaerobiaceae	*Halanaerobium*
			RDP	Firmicutes	Clostridia	Halanaerobiales	Halanaerobiaceae	*Halanaerobium*
		Contig25642	SILVA	Firmicutes	Clostridia	Halanaerobiales		
			RDP	Firmicutes (90)	Clostridia (81)	Halanaerobiales (73)		
		Contig25685	SILVA	Firmicutes	Clostridia	Halanaerobiales	Halanaerobiaceae	*Halanaerobium*
			RDP	Firmicutes (86)	Clostridia (73)			
		Contig26525	SILVA	Firmicutes				
			RDP	Firmicutes (91)	Clostridia (89)	Halanaerobiales (70)		
		Contig27756	SILVA	Firmicutes	Clostridia	Halanaerobiales	Halanaerobiaceae	*Halanaerobium*
			RDP	Firmicutes (84)	Clostridia (78)			
		Contig29794	SILVA	-				
			RDP	Firmicutes (76)				
A	H	Contig01782	SILVA	Firmicutes	Clostridia	Clostridiales	Clostridiaceae	
			RDP	Firmicutes	Clostridia	Clostridiales	Clostridiaceae	*Tindallia*
		Contig02379	SILVA	Firmicutes	Clostridia	Clostridiales	Incertae Sedis XIV	*Anaerobranca*
			RDP	Firmicutes	Clostridia (99)	Clostridiales (98)	Incertae Sedis XIV (88)	*Anaerobranca (88)*
A	I	Contig04714	SILVA	Proteobacteria	Alphaproteobacteria	Rhodobacterales	Rhodobacteraceae	
			RDP	Proteobacteria	Alphaproteobacteria	Rhodobacterales	Rhodobacteraceae	*Rhodobaca (52)*
		Contig08033	SILVA	Proteobacteria	Alphaproteobacteria	Rhodobacterales	Rhodobacteraceae	
			RDP	Proteobacteria	Alphaproteobacteria	Rhodobacterales	Rhodobacteraceae (99)	*Rhodobaca (45)*

* Ass: Assembly used. See Material and Methods section for details

Suppl. Table 2. Continuation

Ass*	Bin	Contig	DB	Phylum	Class	Order	Family	Genus
**A**	**Un****	Contig01627	SILVA	Firmicutes	Clostridia	Natranaerobiales	Natranaerobiaceae	
			RDP	Firmicutes	Clostridia	Natranaerobiales	Natranaerobiaceae	*Natranaerobius*
**B**	**Un****	Contig02658	SILVA	-				
			RDP	Firmicutes	Clostridia	Natranaerobiales	Natranaerobiaceae	*Natronovirga*
		Contig03146	SILVA	Firmicutes	Clostridia	Halanaerobiales	Halanaerobiaceae	*Halanaerobium*
			RDP	Firmicutes	Clostridia	Halanaerobiales	Halanaerobiaceae	*Halanaerobium*
		Contig05582	SILVA	-				
			RDP	Bacteroidetes	Bacteroidia	Bacteroidales (43)		
		Contig05874	SILVA	Proteobacteria	Alphaproteobacteria	Rhizobiales	Bradyrhizobiaceae	*Salinarimonas*
			RDP	Proteobacteria	Alphaproteobacteria	Rhizobiales	Beijerinckiaceae	*Chelatococcus*
		Contig07456	SILVA	-				
			RDP	Firmicutes	Clostridia	Halanaerobiales	Halanaerobiaceae (73)	
		Contig13353	SILVA	Bacteroidetes	Bacteroidia	Bacteroidales	ML635J-40 aquatic group	
			RDP	Bacteroidetes	Bacteroidia	Bacteroidales (65)		

* Ass: Assembly used. See Material and Methods section for details
** Un: Unbinned contigs
