# Supplementary material for: Anaerobic digestion of the microalga Spirulina at extreme alkaline conditions: biogas production, metagenome, and metatranscriptome
Source: Front Microbiol. 2015 Jun 22;6:597. doi: 10.3389/fmicb.2015.00597 (PMC4475827; doi:10.3389/fmicb.2015.00597)
Supplement: Supplementary file 3 [file Table3.PDF]

Supplementary Tables-3

Anaerobic digestion of the microalga *Spirulina* at extreme alkaline conditions: biogas production, metagenome and metatranscriptome

Vimac Nolla-Ardèvol[1*], Marc Strous[1,2,3], Halina E. Tegetmeyer [1,3,4]

[1]Institute for Genome Research and Systems Biology, Center for Biotechnology, Bielefeld University, Bielefeld, Germany.

[2]Department of Geoscience, University of Calgary, Calgary, AB, Canada.

[3]Microbial Fitness Group, Max Planck Institute for Marine Microbiology, Bremen, Germany.

[4] HGF-MPG Group for Deep Sea Ecology and Technology, Alfred Wegener Institute, Helmholtz Centre for Polar and Marine Research, Bremerhaven, Germany

Suppl. Table 3. Metatrancriptome analysis
Alkaline metatranscriptome sequencing data statistics and mapping of mRNA transcripts to bins.

RNA sequencing statistics				
**Sequencing data**	**Bases**	**Reads**	**Mean Read length**	**GC %**
PGM raw data	343 MB	3,341,938	102	49
Post Trimmomatic trimming	241 MB	2,508,254	184	50

Mapping of transcripts					
**Bin**	**Detected CDS**	**Mapped transcripts**	**CDS with mapped reads**	**Functionally annotated CDS***	**Active CDS****
**A**	2,572	66,702	2,226	1,555	1,218
**B**	2,193	45,792	957	708	185
**C**	3,731	33,274	1,101	23	295
**D**	3.479	3,634	536	0	161
**E**	4,537	227,206	3,148	1,950	2,233
**F**	4,100	20,697	1,706	0	505
**G**	8.340	62,874	2,210	0	994
**H**	8,340	23,553	669	382	293
**I**	2,028	6,941	215	130	147

* Total number of CDS which were automatically annotated by GenDB.
**Total number of CDS with mapped transcript with a minimum 1x Coverage and a minimum of 50% covered CDS (See Materials and Methods for details)
