# Supplementary material for: Anaerobic digestion of the microalga Spirulina at extreme alkaline conditions: biogas production, metagenome, and metatranscriptome
Source: Front Microbiol. 2015 Jun 22;6:597. doi: 10.3389/fmicb.2015.00597 (PMC4475827; doi:10.3389/fmicb.2015.00597)
Supplement: Supplementary file 4 [file Table4.PDF]

Supplementary Tables-4

Anaerobic digestion of the microalga *Spirulina* at extreme alkaline conditions: biogas production, metagenome and metatranscriptome

Vimac Nolla-Ardèvol[1*], Marc Strous[1,2,3], Halina E. Tegetmeyer [1,3,4]

[1]Institute for Genome Research and Systems Biology, Center for Biotechnology, Bielefeld University, Bielefeld, Germany.

[2]Department of Geoscience, University of Calgary, Calgary, AB, Canada.

[3]Microbial Fitness Group, Max Planck Institute for Marine Microbiology, Bremen, Germany.

[4] HGF-MPG Group for Deep Sea Ecology and Technology, Alfred Wegener Institute, Helmholtz Centre for Polar and Marine Research, Bremerhaven, Germany

Suppl. Table 4. 16S rDNA accession numbers
Accession numbers of reference 16S rDNA sequences for which in the phylogenetic trees (Figures 5, 6, 7 and Suppl. Fig. 2) only clones and organism names are shown.

Organism name / Clone	Accession
**Cytophaga-Flavobacteria-Bacteroides**	
Salinibacter ruber C37	KF668249.1
Flavobacterium aquatile LMG_4008	NR_118482.1
Sphingobacterium sp.21	NR_074508.1
Gramella forsetii KT0803	NR_074707.1
Anaerophaga thermohalophila Fru22	AJ418048.1
Belliella pelovolcani CC-SAL-25	EU685336.1
Alkaliflexus imshenetskii Z-7010	AJ784993.1
Paludibacter propionicigenes	AB078842.2
Cellulophaga algicola DSM14237	NR_074452.1
Draconibacterium orientale	NR_121783.1
Escherichia coli RREC_I	AF527827.1
CloneML635J-20	AF507861.1
CloneBSA1B-12	AB175366.1
CloneML635J-56	AF507862.1
CloneWN-HWB-154	DQ432348.1
CloneCSS133	JX240684.1
Contig00447 Mesophilic-Reactor	JMBV00000000

Continues in next page

Suppl. Table 4. continuation

Organism name / Clone	Accession
**Clostridiales**	
Orenia sp. 1D4	JQ690693.1
Proteinivorax tanatarense Z-910	JQ904541.1
Tindallia magadiensis Z-7934	NR_026446.1
Halanaerobium hydrogeniformans	NR_074850.1
Clostridium elmenteitii E2SE1	AJ271453.1
Natranaerobius thermophilus JW/NM-WN-LF	NR_074181.1
Alkaliphilus crotonoxidans	AF467248.1
Clostridium difficile 630	NR_074454.1
Thermincola potens JR	NR_074717.1
Thermoanaerobacter sp. X514	NR_074779.1
Natronoanaerobium salstagnum O-M12SP-2	AJ271450.1
Anaerobranca bogoriae	AF203703.1
Natronincola ferrireducens Z-0511	EU878275.1
Halocella cellulosilytica DSM 7362T	X89072.1
Halothermothrix orenii H 168	NR_074915.1
Natronovirga wadinatrunensis JW/NM-WN-LH1	EU338489.2
Arthrospira platensis C1	gi423061881
CloneCSS28	JX240605.1
Clonex216	GU083685.1
CloneCT1C2AC09	JQ427824.1
CloneCSS150	JX240699.1
CloneCSS73	JX240655.1
CloneMAT-CR-H3-B08	EU245154.1
Contig04220 Mesophilic-Reactor	JMBV00000000
Firmicutes-EM1 Mesophilic-Reactor	No Accession #
**Alphaproteobacteria**	
Rhodobaca bogoriensis SLB	EU908048.1
Chelatococcus sp. J-9.1	FR774565.1
Rhodobacter sp. R-8	AY914074.1
Roseinatronobacter sp. MOL1.10	KJ486297.1
Rhodobaca barguzinensis VKM_B-2406	NR_044285.1
Rhodobacter sphaeroides 2.4.1	NR_074171.1
Methylobacterium extorquens AM1	NR_074138.1
Salinarimonas rosea YIM-YD3	NR_116487.1
Bacillus subtilis	X60646.1
CloneSA_118_	JQ739039.1
CloneTUM-Mbac-MR4-B1-KC14	EU812964.1
CloneQEEA3DF04	CU918797.1
CloneML602J-43_	AF507829.1
CloneTX4CB_152	FJ153021.1

Continues in next page

Suppl. Table 4. Continuation

Organism name / Clone	Accession
**Methanogens**	
Methanoculleus bourgensis MS2	NR_042786.1
Methanosarcina mazei zm-15	KF360023.1
Methanocalculus sp. AMF2	HM053969.1
Methanocalculus sp. AMF-Bu2	JQ724113.1
Methanocalculus halotolerans	NR_041843.1
Methanocalculus natronophilus strain Z-7105	NR_118529.1
Methanobacterium oryzae	AF028690.2
Methanospirillum lacunae	AB517986.1
Methanosalsum zhilinae DSM4017	NR_102894.1
Methanobacterium alcaliphilum NBRC105226	NR_112910.1
Methanocaldococcus sp. FS406-22	NR_074228.1
Uncultured *Methanohalophilus sp*. cloneGNA03F08	EU731585.1
Uncultured *Methanococcoides sp*. cloneET5-1H2	EU585970.1
Uncultured *Methanocalculus sp*. cloneNRA1	HM041902.1
Uncultured *Methanocalculus sp*. cloneD008023G07	GU179434.1
Methanopyrus kandleri AV19	NR_074539.1
CloneARC204	JN185089.1
CloneFL-52	DQ089009.1
CloneWN-FWA-130	DQ432522.1
CloneNRP-N	AB243805.1
Clone61ArcR3	JF421670.1
CloneA161	FJ205789.1
CloneAS22	EU358672.1
Contig11126 Mesophilic-Reactor	JMBV00000000
