# Supplementary material for: Anaerobic digestion of the microalga Spirulina at extreme alkaline conditions: biogas production, metagenome, and metatranscriptome
Source: Front Microbiol. 2015 Jun 22;6:597. doi: 10.3389/fmicb.2015.00597 (PMC4475827; doi:10.3389/fmicb.2015.00597)

Supplementary Figure-1

Anaerobic digestion of the microalga *Spirulina* at extreme alkaline conditions: biogas production, metagenome and metatranscriptome

Vimac Nolla-Ardèvol[1*], Marc Strous[1,2,3], Halina E. Tegetmeyer [1,3,4]

[1]Institute for Genome Research and Systems Biology, Center for Biotechnology, Bielefeld University, Bielefeld, Germany.

[2]Department of Geoscience, University of Calgary, Calgary, AB, Canada.

[3]Microbial Fitness Group, Max Planck Institute for Marine Microbiology, Bremen, Germany.

[4] HGF-MPG Group for Deep Sea Ecology and Technology, Alfred Wegener Institute, Helmholtz Centre for Polar and Marine Research, Bremerhaven, Germany

Suppl. Figure 1. Detection of aggregates/granules

In order to determine the presence of aggregates/granules in the alkaline reactors, microscopic pictures were taken from sludge samples of each different period of the three alkaline reactors Alk-HRT, Alk-OLR and Alk-Opt. Fresh sludge from a laboratory scale non-alkaline anaerobic reactor inoculated with sludge from a waste water treatment plant and fed solely with starch was used as a positive control (WW-PA). As the alkaline sludge samples used for this analysis were kept at -20ºC and were subsequently thawed to take the microscopic pictures, a second positive control was used (T-WW-PB). This consisted of frozen-thawed sludge obtained from the same non-alkaline anaerobic reactor as the positive control WW-PA. Additionally, fresh sludge from a running alkaline reactor (pH~10; 2.0 M Na^+) fed with *Spirulina* was used as a third control (Alk-Fresh).

Microscopic pictures of **(A)** Controls; **(B)** Alk-HRT; **(C)** Alk-OLR, and **(D)** Alk-Opt reactors.

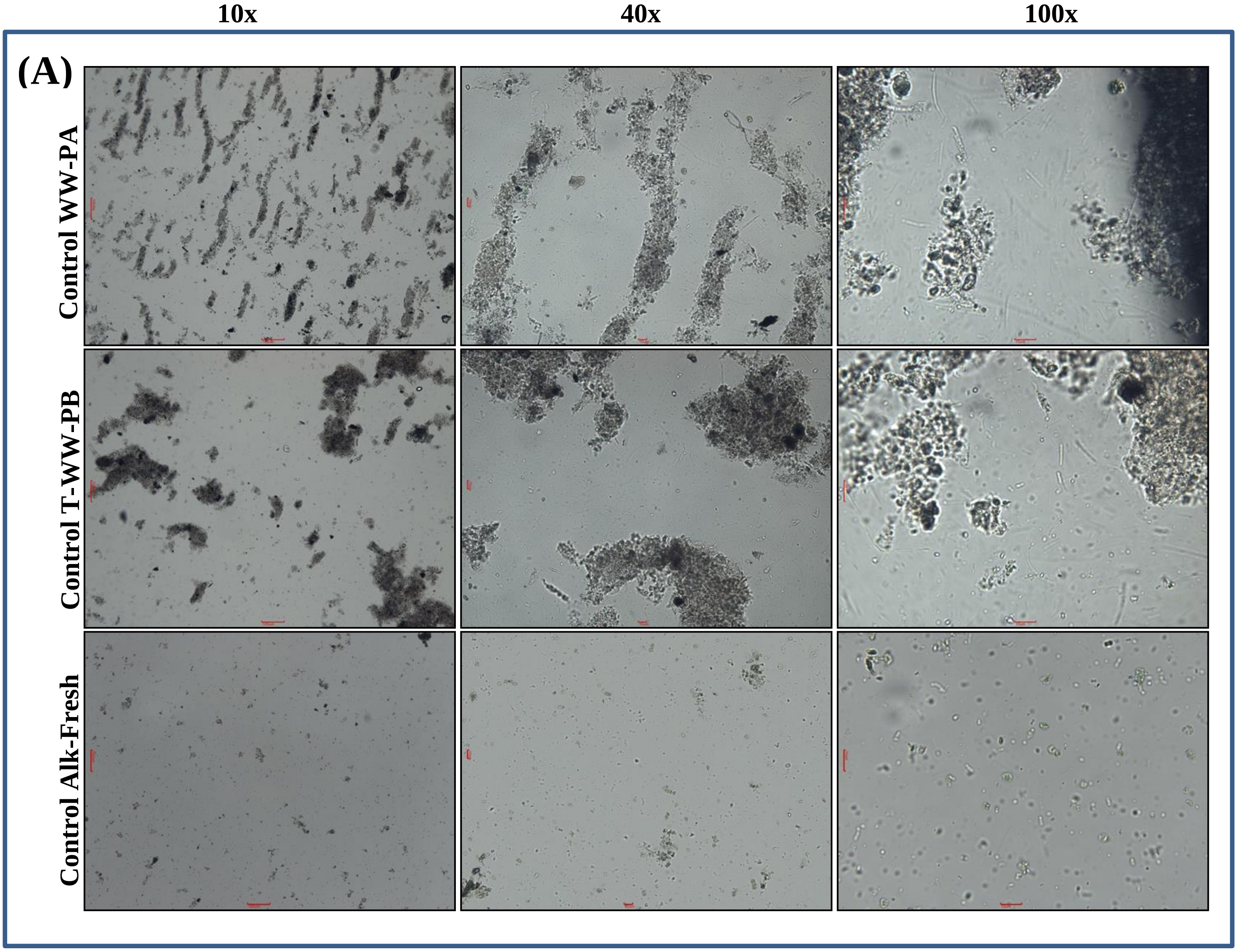
10x
40x
100x
(A)
Control WW-PA
Control T-WW-PB
Control Alk-Fresh

Suppl. Figure 1. Detection of aggregates/granules (Continuation)

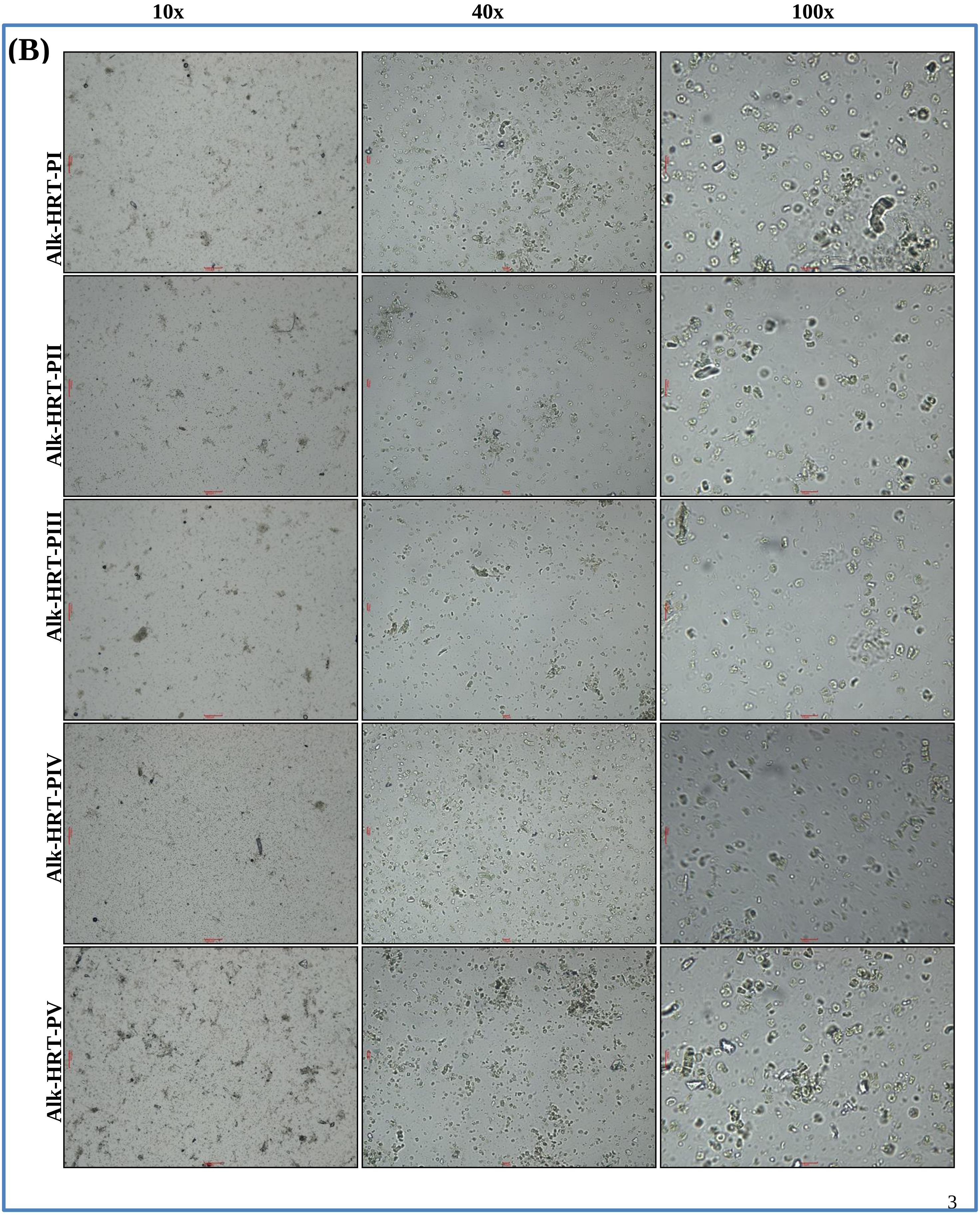

Suppl. Figure 1. Detection of aggregates/granules (Continuation)

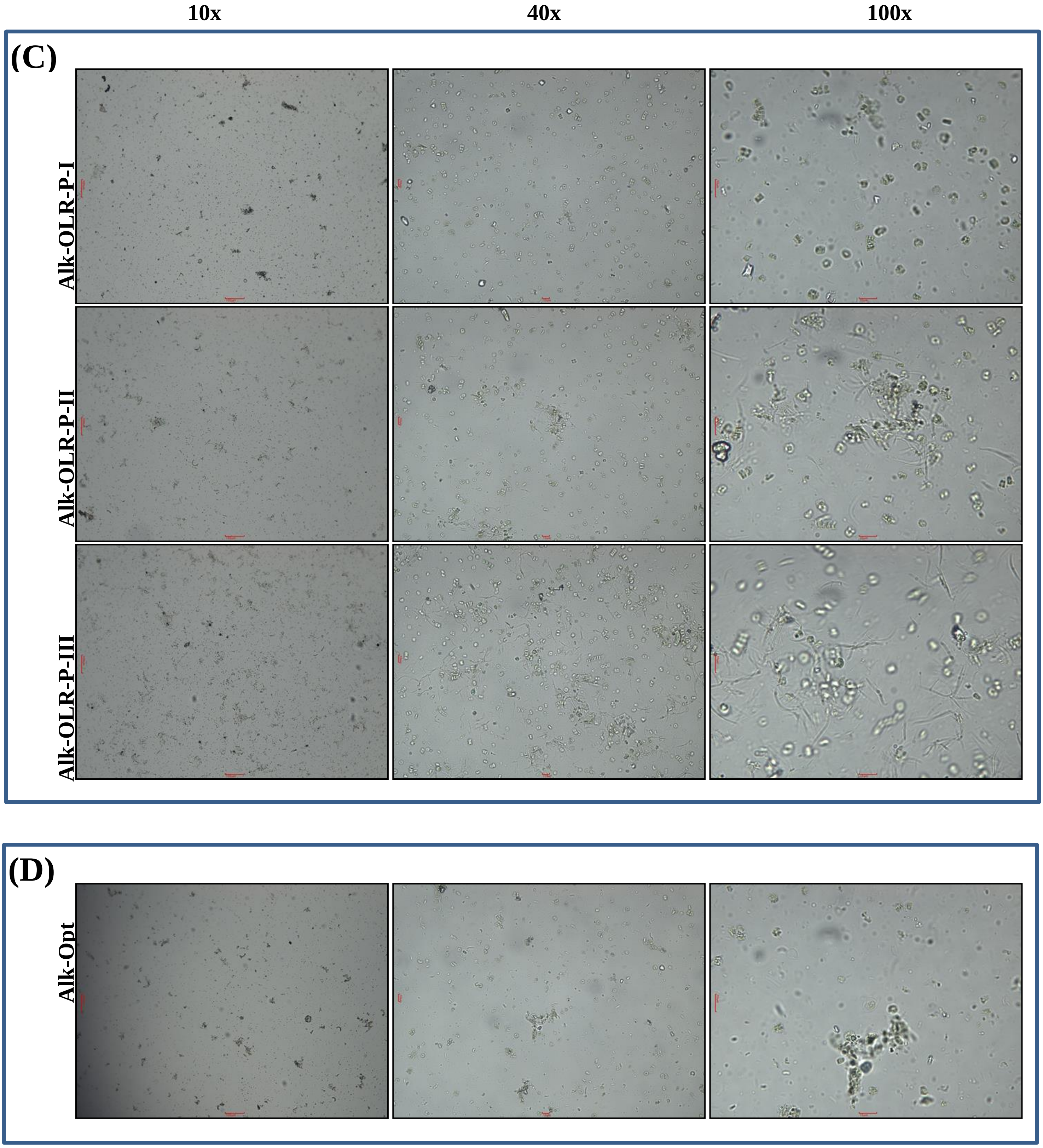

Supplement: Supplementary file 5 [file Image1.PDF]
