# Supplementary material for: Anaerobic digestion of the microalga Spirulina at extreme alkaline conditions: biogas production, metagenome, and metatranscriptome
Source: Front Microbiol. 2015 Jun 22;6:597. doi: 10.3389/fmicb.2015.00597 (PMC4475827; doi:10.3389/fmicb.2015.00597)
Supplement: Supplementary file 6 [file Image2.PDF]

Supplementary Figure-2

Anaerobic digestion of the microalga *Spirulina* at extreme alkaline conditions: biogas production, metagenome and metatranscriptome

Vimac Nolla-Ardèvol[1*], Marc Strous[1,2,3], Halina E. Tegetmeyer [1,3,4]

[1]Institute for Genome Research and Systems Biology, Center for Biotechnology, Bielefeld University, Bielefeld, Germany.

[2]Department of Geoscience, University of Calgary, Calgary, AB, Canada.

[3]Microbial Fitness Group, Max Planck Institute for Marine Microbiology, Bremen, Germany.

[4] HGF-MPG Group for Deep Sea Ecology and Technology, Alfred Wegener Institute, Helmholtz Centre for Polar and Marine Research, Bremerhaven, Germany

Suppl. Figure 2. 16S rDNA Alphaproteobacteria phylogenetic tree
16S rDNA phylogenetic tree of the contigs assigned to members of the Alphaproteobacteria class by the RDP and SILVA classifiers. Although the binning was performed with contigs of assembly A, the tree also includes those contigs that were obtained from assembly B and were not assembled in assembly A. Minimum contig length of 1000bp. Colored: sequences obtained from metagenomic reads. Assignment to Metawatt bins and percentage of bin abundance is indicated if applicable. Reference sequences in **bold**: top hits in blast search against NCBI reference RNA sequences database; **bold + underlined:** top hits in blast search against NCBI non-redundant nucleotide collection. Additional reference sequences tree represent genera detected in other alkaline environments or anaerobic digesters. 16S rRNA sequence of *B. subtilis* was chosen as outgroup. Bootstrap values at nodes are obtained from 500 replicates and are only shown for branches with at least 50% support (values > 49.9). The scale bar represents 0.01 nucleotide substitutions per site. Accession numbers of reference sequences are available in Suppl. Table 4.

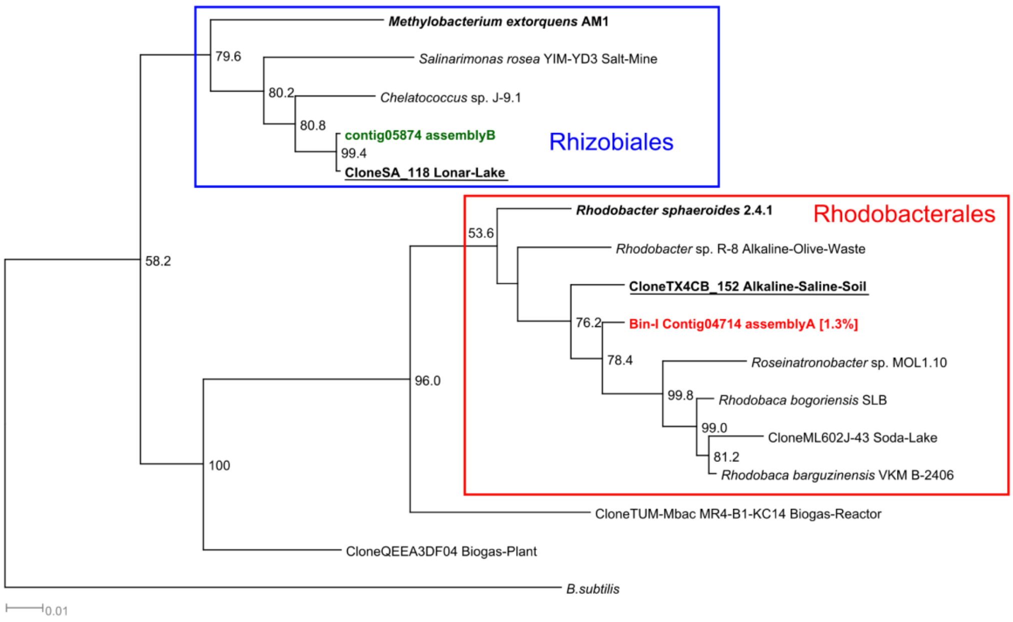
Methylobacterium extorquens AM1
79.6
Salinarimonas rosea YIM-YD3 Salt-Mine
80.2
Chelatococcus sp. J-9.1
80.8
contig05874 assemblyB
99.4
CloneSA_118 Lonar-Lake
Rhizobiales
Rhodobacter sphaeroides 2.4.1
Rhodobacterales
53.6
Rhodobacter sp. R-8 Alkaline-Olive-Waste
58.2
CloneTX4CB_152 Alkaline-Saline-Soil
76.2
Bin-I Contig04714 assemblyA [1.3%]
78.4
Roseinatronobacter sp. MOL1.10
96.0
99.8
Rhodobaca bogoriensis SLB
99.0
CloneML602J-43 Soda-Lake
100
81.2
Rhodobaca barguzinensis VKM B-2406
CloneTUM-Mbac MR4-B1-KC14 Biogas-Reactor
CloneQEEA3DF04 Biogas-Plant
B.subtilis
0.01
